# Microbial communities reveal niche partitioning across the slope and bottom zones of the challenger deep

**DOI:** 10.1111/1758-2229.13314

**Published:** 2024-07-31

**Authors:** Aoran Hu, Weishu Zhao, Jing Wang, Qi Qi, Xiang Xiao, Hongmei Jing

**Affiliations:** ^1^ State Key Laboratory of Microbial Metabolism, School of Life Sciences and Biotechnology Shanghai Jiao Tong University Shanghai China; ^2^ International Center for Deep Life Investigation (IC‐DLI) Shanghai Jiao Tong University Shanghai China; ^3^ School of Oceanography Shanghai Jiao Tong University Shanghai China; ^4^ SJTU Yazhou Bay Institute of Deepsea Sci‐Tech Yongyou Industrial Park Sanya China; ^5^ Southern Marine Science and Engineering Guangdong Laboratory (Zhuhai) Zhuhai Guangdong China; ^6^ Institute of Deep‐Sea Science and Engineering Chinese Academy of Sciences Sanya China

## Abstract

Widespread marine microbiomes exhibit compositional and functional differentiation as a result of adaptation driven by environmental characteristics. We investigated the microbial communities in both seawater and sediments on the slope (7–9 km) and the bottom (9–11 km) of the Challenger Deep of the Mariana Trench to explore community differentiation. Both metagenome‐assembled genomes (MAGs) and 16S rRNA amplicon sequence variants (ASVs) showed that the microbial composition in the seawater was similar to that of sediment on the slope, while distinct from that of sediment in the bottom. This scenario suggested a potentially stronger community interaction between seawater and sediment on the slope, which was further confirmed by community assembly and population movement analyses. The metagenomic analysis also indicates a specific stronger potential of nitrate reduction and sulphate assimilation in the bottom seawater, while more versatile nitrogen and sulphur cycling pathways occur on the slope, reflecting functional differentiations among communities in conjunction with environmental features. This work implies that microbial community differentiation occurred in the different hadal niches, and was likely an outcome of microbial adaptation to the extreme hadal trench environment, especially the associated hydrological and geological conditions, which should be considered and measured in situ in future studies.

## Graphical abstract

The physical–chemical characteristics related to depth across the slope and bottom, along with the distinct hydrology between seawaters and sediments, influence the microbial communities in the challenger deep of the Marianas trench. This leads to niche differentiation in community composition and metabolic functions, which is a result of niche adaptation.

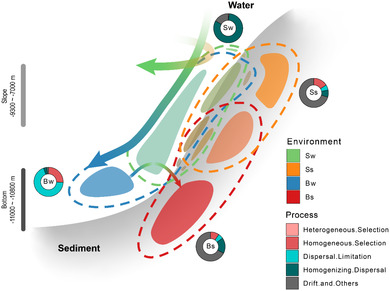

## INTRODUCTION

The Mariana Trench encompasses the deepest point on the surface of Earth, the Challenger Deep, at a water depth of ~11 km below sea level (b.s.l.) (Du et al., 2021). Hadal zone, which refers to marine habitats with a depth greater than 6000 m, is far beyond the reach of sunlight. Challenger Deep is the deepest among all hadal zones and is under high hydrostatic pressure (HHP) that increases up to ~115 MPa (Xiao et al., 2021). In general, the Mariana Trench is located underneath the oligotrophic ocean and lacks a substantial influx of terrestrial organic matter due to its great distance from the shore (Li et al., 2021; Luo et al., 2017), with organic carbon influxes as low as 0.55 g C m^−2^ year^−1^ (Stewart & Jamieson, 2018; Xu et al., 2018). However, the V‐shaped topology of the Challenger Deep leads to the accumulation of particulate organic matter (POM) and heavy metals (e.g., As and Se), resulting in higher concentrations of nutrients and higher benthic microbial activity at the bottom of the Challenger Deep than on adjacent slopes, but both are higher than those in the abyssal plain (Glud et al., 2013; Wenzhöfer et al., 2016; Zhou et al., 2022).

Extreme environmental conditions of the Challenger Deep harbour a special ecosystem dominated by microbes in both seawater and sediments (Du et al., 2021). Dynamic geophysical and oceanographic processes possibly cause exchanges between sediments and seawater (especially near‐benthic seawater) via sediment resuspension events, which may further affect the composition and distribution of microbial communities (Liu et al., 2019; Nunoura et al., 2015). However, the sediment and seawater in the hadal zone were generally considered separated ecosystems in previous studies. In the Challenger Deep (where the bottom flow was usually considered very slow) (Jiang et al., 2021), it is unclear whether the seawater‐sediment exchange still occurred and how it impacted the spatial distribution characteristics of microbes living there. The steep slope, narrow geomorphology, dynamic ocean circulation, and seismic activities of the Challenger Deep suggest potential exchanges across different locations within the Challenger Deep (Nunoura et al., 2016; Shimabukuro et al., 2022; Stewart & Jamieson, 2018; Zhu et al., 2019). In addition, the remixing of POM with near‐benthic seawater may not only intensify carbon turnover but also affect the supply of electron donors (e.g., various carbon resources, ammonia and nitrite) and acceptors (e.g., oxygen, nitrate, and sulphate) (Nunoura et al., 2015), shifting potential element cycling. However, the exchange of the microbial community either within seawater or between seawater and sediment in the Challenger Deep remains unclear. The extent to which this exchange influences the turnover of microbially mediated organic carbon and the cycling of elements in the hadal zone also remains unexplored. On the other hand, the physical exchange also can transfer microbes into a different niche, thus affecting community assembly and composition (Bakker et al., 2004). Niche differentiated populations can exhibit preferences that reflect the habitat. These preferences can affect nutrient cycling and, the stability of the community, and eventually could result in speciation or adaptive radiation (Miller et al., 2023). Factors influencing niche differentiation range from environmental variables such as temperature, HHP, and POM availability to biotic interactions including competition, cooperation, and symbiosis. Unravelling the complex interactions between these factors is crucial for understanding the mechanisms driving microbial community assembly and ecosystem dynamics in Challenger Deep. Microbial community composition and functional potentials may provide some clues.

Due to the challenges of sampling techniques and in situ measuring methods at high pressures in deep‐sea areas (especially the hadal zone), previously microbial community studies were typically designed to investigate seawater and sediment separately, and there is still limited comparative research on sediment and near‐benthic seawater samples in the same area of the ocean. Seawater‐based studies have mostly focused on the microbial carbon cycle. In seawater, the dominant chemolithoautotrophs are generally replaced by heterotrophs with increasing water depth from the surface to the hadal zone (Nunoura et al., 2015; Tian et al., 2018). At water depths greater than 6 km functional marker gene and metagenome data have indicated the essential roles of predominant heterotrophs in recycling macromolecules and utilizing various carbon sources (e.g., peptides, carbohydrates, hydrocarbons, aromatic compounds) (Liu et al., 2019; Tian et al., 2018). An abrupt increase in the abundance of hydrocarbon‐degrading bacteria was observed at depths over 10 km in the water column of the Challenger Deep and represents the highest proportion of microbiological communities observed in any natural environment on Earth (Liu et al., 2019; Nunoura et al., 2015). The niche separation of nitrifiers varies with water depth in the vertical water column (Nunoura et al., 2015). Sediment‐based studies, on the other hand, revealed possible differences in nitrogen cycling at different locations at various water depths in the Challenger Deep. Geochemistry, functional marker genes, and metagenome data suggest a potentially complex nitrogen cycle in the Challenger Deep sediments, including the co‐occurrence of aerobic nitrification and anaerobic denitrification as well as the potential occurrence of the anammox process (Gao et al., 2019; Jing et al., 2022; Liu & Peng, 2019; Nunoura et al., 2018; Zhou et al., 2022). The community composition of benthic microbial communities mediating the carbon‐nitrogen cycle varied with a location in the sediments at the slope or bottom of the Challenger Deep (Chen et al., 2021; Jing et al., 2022; Zhou et al., 2022). Growing evidence has shown prominent nitrate utilisation in hadal sediments (Hiraoka et al., 2020; Liu & Peng, 2019; Thamdrup et al., 2021; Zhou et al., 2022), and the metabolic potential of nitrogen transformations is ubiquitous among hadal microbial communities (Chen et al., 2021; Nunoura et al., 2013; Zhou et al., 2022). Nevertheless, the relationship between microbial communities in sediments and seawater has rarely been investigated in hadal environments. The only study conducted at the bottom of the Challenger Deep suggested distinct microbial community compositions between near‐bottom seawater and sediments (Nunoura et al., 2018). A systematic comparison of microbial community composition and metabolic potential between seawater and sediment of the Challenger Deep has not yet been reported.

In this study, we obtained seawater samples from 10 sites on the slope and bottom of the Challenger Deep and compared them with sediment samples collected at similar water depths from 15 sites in previous studies. A total of 357 dereplicated metagenome‐assembled genomes (MAGs) with 95% nucleotide identity from 33 metagenomic samples and 4734 amplicon sequence variants (ASVs) based on 16S rRNA gene sequencing of 13 samples were used to profile the microbial community diversity, community composition, and metabolic potentials in the near‐benthic seawater and sediments of both the bottom and slope. We further constructed a MAG‐based framework to quantify the main driving forces (i.e., homogeneous selection, heterogeneous selection, homogenizing dispersal, dispersal limitation, drift) of community assemblies and to calculate population movement. Both MAG and 16S rRNA analysis quantitatively reflect the effect of exchange on the construction, succession and distribution of the hadal microbial community. This study was conducted to investigate the potential niche differentiation within the seawater and between the seawater and sediment at both the bottom and slope of the Challenger Deep, and how these interaction processes impact microbial communities and their metabolic capabilities in elemental cycling.

## EXPERIMENTAL PROCEDURES

### Sampling, preprocessing, and sequencing

**FIGURE 1:**
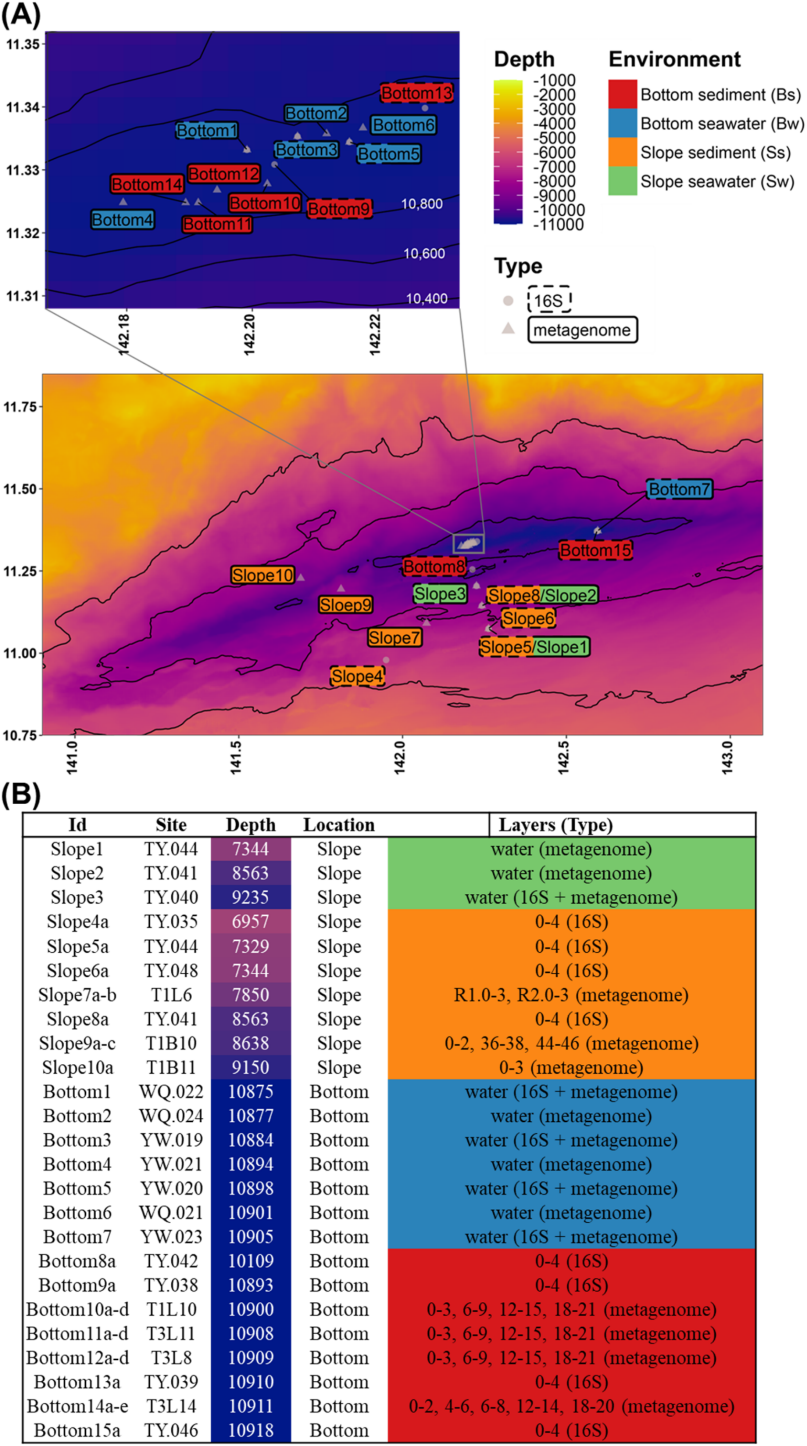
Sampling sites, depth, locations, and grouping. Seawater samples are collected from the slope and bottom of the Mariana Trench near the seabed in the hadal zone; sediment samples are referenced from Zhou et al. (2022) and Wang et al. (2022).

Ten seawater samples were collected aboard the R/V ‘Tansuo‐01’ using Niskin bottles from stations on the bottom axis and slope of the Challenger Deep in the Mariana Trench in three dives of the ‘Tianya’ lander, four dives of the ‘Yuanwei’ lander and three dives of the ‘Wanquan’ lander during the TS‐09 cruise in September and October 2018 (Figure 1B). Samples from stations TY044 (sample id Slope1) and TY041 (sample id Slope2) were filtered directly through 0.22 μm polycarbonate membranes (142 mm, Millipore), and the other eight samples were continuously filtered through 3 and 0.22 μm polycarbonate membranes aboard for higher efficiency. No significant difference between filter methods was obtained in further analysis. All filters were frozen at −80°C immediately until further analysis with the addition of RNAlater™ Stabilization Solution (Thermo Scientific, Wilmington, DE, USA). In situ, hydrographical parameters (e.g., salinity, temperature, and depth) were measured by a conductivity‐temperature‐depth (CTD) rosette system (Sea‐Bird Electronics) during the sampling processes. Genomic DNA of seawater samples was extracted from the frozen 0.22 μm filters with a PowerSoil DNA Isolation Kit (Qiagen, Germantown, USA) following the manufacturer's protocol. The concentration of DNA was quantified by a Qubit DNA Assay Kit with a Qubit 2.0 fluorometer (Invitrogen, Carlsbad, CA, USA), and the quality was checked by gel electrophoresis. The sequencing library was prepared using the KAPA HyperPrep Kit (Roche, Shanghai, China). Shotgun sequencing was performed on an Illumina NovaSeq 6000 Platform PE150 (Illumina, San Diego, CA, USA) with 150 bp paired‐end reads. As a comparison of these seawater samples, raw reads of 23 metagenomes of sediment samples from seven stations in the Mariana Trench were collected from a previous study (Zhou et al., 2022). For 16S rRNA gene amplicon sequencing, the V3‐V4 region of the 16S rRNA gene was amplified using the following primer pair: Fwd, CCTACGGGNBGCASCAG; Rev, GACTACNVGGGTATCTAATCC (Takahashi et al., 2014). Paired‐end sequencing of the amplicons was then performed with an Illumina HiSeq PE250 sequencer (Novogene Co., Ltd., www.novogene.com). For comparison, 16S rRNA V3V4 sequencing data from eight sediment samples were collected from a previous study (Wang et al., 2022). Detailed station information for both seawater and sediments used in this study is shown in Figure 1.

### Binning and MAG‐level analysis of metagenomic data

Raw reads of metagenomic data of both seawater samples collected in this study and sediment samples collected in the previous study (Zhou et al., 2022) were analysed together in this study. All these metagenomic raw reads were filtered using sickle (version 1.33) in PE mode with parameters (‐‐length‐threshold 90 ‐t sanger ‐g) (najoshi, 2022). The quality of filtered reads was checked with fastQC (v0.11.4) and multiQC (version 1.9) (Ewels et al., 2016). Metagenomic contigs were also assembled from clean reads using megahit (v1.2.9) with default parameters (Li et al., 2015) for each sample. Shotgun sequencing of the 10 near‐benthic seawater samples yielded 59,679,900 clean raw read pairs after being trimmed and filtered, accounting for 98.36% of the raw reads. Assembly of those 33 metagenomes generated 11,162,542 contigs (average length of 1127 bp).

Clean reads were mapped to contigs assembled from the same sample using bbmap.sh (Bushnell, 2014). The coverage of contigs in each sample was calculated by jgi_summarize_bam_contig_depths from metabat2. Metagenomic binning and refinement were performed on contigs using a combination of metabat2 (version 2:2.15), maxbin2 (version 2.2.7), concoct (version 1.1.0), checkm (version 1.1.3), and DASTool (version 1.1.2) (Alneberg et al., 2014; Kang et al., 2019; Parks et al., 2015; Sieber et al., 2018; Wu et al., 2016) for each sample. Contig coverage was calculated using bbmap.sh (last modified on 13 February 2020) by remapping clean reads to contigs (Bushnell, 2014). Metabat2 was run with nine combinations of two parameters (‐‐maxP with 60, 75, 90, and ‐‐minS with 60, 75, 90). Maxbin2 was used with two combinations of parameters (‐markerset with 107 and 40). Concoct was used with parameters (‐r 150 ‐s 599 ‐‐no_original_data). Binning results (draft genomes) from the same sample of the above 12 methods were then combined and refined using DASTool. A draft genome with contamination >10% (could not fulfil the MIMAG middle‐quality threshold; Bowers et al., 2017) was detected as the only genome containing the nitrogen fixation genes *nifDK*. As there have been no reports on the nitrogen‐fixing population in the trenches, we hoped to gain a deeper understanding of this function based on this finding. Therefore, we manually curated the genome, reduced the level of contamination, and finally obtained a draft genome containing the *nifDK* genes in our study. This genome belongs to the known nitrogen fixation genus *Bradyrhizobium* in the class *Alphaproteobacteria*. Next, binning results from all samples were collected, resulting in 792 draft genomes with qualities that meet the middle‐quality threshold (≥50% completeness and ≤10% contamination), among which 289 meet the high‐quality threshold (≥90% completeness and ≤5% contamination) according to MIMAG (Bowers et al., 2017). To represent a species‐level clade across samples in the study, dRep (v3.0.0) was used with parameters (‐comp 50 ‐con 10 ‐pa 0.9 ‐sa 0.95 ‐N50W 2) to select representative draft genomes for each species‐level (95% ANI) genome cluster among these 792 middle‐ or high‐quality draft genomes (Olm et al., 2017), and the resulting 357 genomes were hereafter referred to as MAGs. GTDB‐Tk (v1.6.0) was used to taxonomically classify representative MAGs of all clusters with the GTDB database release 202 (Chaumeil et al., 2019; Parks et al., 2020).

### MAGs abundance and phylogenomic analysis

Reads were mapped to genes of representative MAGs by bwa with methods provided by coverM, and the relative abundance of MAGs for population composition was calculated by coverM with parameters (‐m relative_abundance ‐‐min‐read‐aligned‐length 50 ‐‐min‐read‐percent‐identity 0.99 ‐‐min‐covered‐fraction 0.1 ‐‐proper‐pairs‐only) (version 0.6.1) (Woodcroft, 2024). Reads mapped to genes in representative MAGs were counted by featureCounts (version 2.0.1) (Liao et al., 2014).

A phylogenetic tree of reconstructed representative MAGs was constructed using the protein sequences of 40 universal gene markers. For each phylum that occurred in MAGs in this study, one MAG was randomly selected from all classes as reference genomes. Genes of each genome were predicted by Prodigal as described previously (Lv et al., 2022). fetchMGs (v1.2) was used to extract 40 conserved single‐copy genes of prokaryotes from each genome (Sorek et al., 2007), and genomes with less than 20 extracted marker genes were filtered. Marker genes were aligned by MAFFT (v7.487) with parameters (‐‐maxiterate 1000 ‐‐localpair) separately and concatenated to trim by trimAl (v1.4.rev15) with automated1 mode. IQ‐TREE (version 2.0.3) was used to construct the tree with parameters (‐m MFP ‐B 1000 ‐‐bnni ‐T AUTO). All trees were further polished and visualized using the Interactive Tree Of Life (iTOL) v5 (Letunic & Bork, 2021).

### Analysis of 16S amplicon data

The sequencing reads of the 16S rRNA amplicon was analysed using the QIIME2 platform (v2023.5) (Bolyen et al., 2019). Briefly, the adaptors and primers were trimmed with Cutadapt (Martin, 2011). Next, the paired‐end reads were merged and denoised into ASVs using DADA2 (Callahan et al., 2016). The final ASV table was rarified to retain 38,000 reads per sample to omit bias from uneven sequencing depths (McDonald et al., 2012; McKinney, 2010; Weiss et al., 2017). Next, the ASVs were classified using sklearn against the training set from SILVA database version 138.1 (Bokulich et al., 2018; Pedregosa et al., 2011; Pruesse et al., 2007; Quast et al., 2013; Rognes et al., 2016). Finally, representative sequences of the ASVs were aligned by MAFFT before they were input into FastTree 2 to construct a phylogenetic tree (Katoh & Standley, 2013; Price et al., 2010). A total of 4735 ASVs from 16S rRNA amplicon sequencing were identified and rarefied to 38,000 reads per sample. Downstream statistical analyses were performed using R version 4.1.3 (R Core Team, 2022) and the R packages vegan (v2.5.7) and ecodist (v2.0.7) (Goslee & Urban, 2007; Oksanen et al., 2020; R Core Team, 2020). Data manipulation and visualisation were then completed in the tidyverse (v1.3.1) and ggplot2 (v3.3.5) packages (Wickham, 2016; Wickham et al., 2019).

### Quantification of assembly processes

We applied a framework to quantitatively infer community assembly mechanisms by phylogenetic bin‐based null model analysis (iCAMP); the community assembly mechanisms of genome‐level microbial communities were determined by using the ‘iCAMP’ R package (Ning et al., 2020), which includes three steps: (i) obtaining phylogenetic bins with adequate phylogenetic signals; (ii) calculating ecological processes governing each bin; and (iii) statistical analysis. 16S amplicon data was used for quantification. Since the mapping rate of MAGs against raw reads was relatively high (average 53.4%), we also used the relative abundance of MAGs in each sample and the phylogenetic tree of MAGs as two input files with parameters in iCAMP (ds = 0.2, rand.time = 300, nworker = 64, memory.G = 500, bin.size.limit = 24, sig.index = ‘Confidence’, detail. null = TRUE).

### Estimation of net microbial population movement

The estimations of net microbial population movement between samples were performed by comparing the relative abundance of species‐level MAGs after dereplication following the calls in the dRep software (Olm et al., 2017). The relative abundance of MAGs here was calculated by coverM with similar parameters (except ‐m RPKM). The original sample of the species‐level MAGs was proxied as the sample containing the representative MAG. We made this estimation based on the concept that microbial species would derive most often from the sample with the highest coverage, wherein its representative genome was most likely to be assembled. This was inspired by the Tara Oceans Viromes study (Brum et al., 2015). Consistent with the results in the reference, we also observed that the MAGs generally had the highest abundance in their original samples. For each pair of samples, the microbial movement flux from sample A to B (*f*_AB_) was calculated as the average relative abundance of MAGs originating from sample A in sample B. We then defined the net flux of microbial movement (*F*_AB_) and the mixture index of samples A and B (*M*_AB_) shown in Equations (1) and (2) as follows:

(1) $$F_{\mathrm{AB}}=f_{\mathrm{AB}}-f_{\mathrm{BA}}$$

(2) $$M_{\mathrm{AB}}=\frac{f_{\mathrm{AB}}+f_{\mathrm{BA}}}{2}$$

The absolute value of *F*_AB_ denotes the magnitude of movement. The sign of *F*_AB_ indicates the direction (positive *F*_AB_ means net microbial movement from sample A to B, and negative *F*_AB_ implies the opposite). For the sample pairs whose *F*_AB_ was close to zero, it is possible that these samples were isolated, such as the sediment samples that were far apart. On the other hand, it was also possible that the samples had equally high flux into each other, such as the seawater and sediment samples from the neighbouring sites. These two cases could be further distinguished by the *M*_AB_ value. To assess the statistical significance of the estimations, we performed permutations (*n* = 9999) on the original samples of MAGs and recalculated the corresponding *F*_AB_ and M_AB_ values. As a result, a pair of samples was identified as ‘Isolated’ when the *M*_AB_ value was lower than 95% of the permutated ones; ‘Transmitted’ when the *F*_AB_ value was higher than 95% of the permutated cases and this pair of samples was not ‘Isolated’; or ‘Mixed’ when the *M*_AB_ value was higher than 95% of the permutated ones and this pair of samples was not ‘Transmitted’. The remaining pairs of samples that could not be distinguished from random permutation results were identified as ‘Others’.

### Gene annotation

Genes were predicted by Prodigal (V2.6.3) in single mode (Hyatt et al., 2010) and dereplicated with MMseq2 (version 13.45111) for 95% identity (Steinegger & Söding, 2017). Representative genes were then annotated by Mantis to assign KEGG KO to genes with the default database (Queirós et al., 2021) (Table S4). We also used pathways and modules from KEGG brite (ko00002) for further analysis.

### Statistical analysis

The prevalence of each taxon in different samples was estimated by the relative abundance of MAGs. Mapped reads of genes are grouped by KO and KEGG modules for each sample (Coelho et al., 2022). Distances between samples were calculated by *vegdist* in the R package *vegan* using the Jaccard and Bray–Curtis distances (Oksanen et al., 2020). Genes per million reads (GPM) was used to normalize gene abundance data for metagenomic comparison, which is the method equivalent to transcripts per million (TPM) in transcriptomes (He et al., 2022; Liu et al., 2022; Zhou et al., 2022). Briefly, reads mapped to genes were normalized by gene length and total mapped number following the formula below:

$$\mathrm{GP}M_{k}=\frac{r_{k}/l_{k}}{\sum \left(r_{i}/l_{i}\right)}\times 10^{6}$$

where $r_{i}$ and $l_{i}$ refer to the reads mapped to the gene i and the length of the gene i, respectively. The significant difference between different environments was calculated using the PERMANOVA method in the R package *Vegan*. The significance of the gene abundance in MAGs between seawater and sediment samples at either the bottom or slope was calculated first using the *Wilcox* test and then adjusted by Bonferroni and Hochberg correction in R (Ahlmann‐Eltze & Patil, 2021; Benjamini & Hochberg, 1995). Co‐occurrence networks of genes were constructed based on their presence/absence in MAGs of different environments based on binary Jaccard distance. Edges in the network represent a co‐occurrence level higher than 0.3.

## RESULTS

### Description of samples and environmental parameters

During the TS‐09 cruise in September and October 2018, seawater samples were collected at 10 stations (seven at the bottom and three at the southern slope) in the Challenger Deep (Figure 1, Methods); these sampling sites varied in water depth and hydrostatic pressure, ranging from 7 to 11 km b.s.l. (75 to 115 MPa) during the ‘TS‐09’ cruise aboard the R/V ‘Tansuo‐01’. The temperature of these sampling sites ranged from 1.8 to 2.6°C and varied with water depth, while salinity was approximately 34.7 practical salinity units (psu) or ~3.5% (w/v). The concentration of nitrogen species was measured in the seawater samples and compared with those in sediment samples from similar stations that were reported in previous studies (Hiraoka et al., 2020; Liu & Peng, 2019; Zhou et al., 2022). The concentrations of ammonium and nitrite in the seawater samples were comparable to those in sediment samples at similar water depths at the bottom of the Challenger Deep, where 1.9–3.9 μM ammonium was measured in the seawater samples and 0.8–6.3 μM ammonium was reported in the surface layer (0–2 cm) of the sediment samples. All 10 samples were sequenced for metagenomic data, and five of them were further sequenced for 16S rRNA amplicon data. In addition, eight 16S rRNA sediment samples from the same cruise and 23 metagenomes at seven sediment sampling stations (four at the bottom and three on the slope) in the Challenger Deep from previous studies were collected for comparison (Figure 1 A,B) (Wang et al., 2022; Zhou et al., 2022). All the sediment samples were collected from the surface aerobic layer of the seabed. The 33 samples were divided into four groups according to their original sampling sites: slope seawater, slope sediments, bottom seawater, and bottom sediments.

### Comparison of the community structures between seawater and sediment on the slope and bottom

The relative abundances of MAGs and ASV were used to describe the community structure. Using nonmetric multidimensional scaling (NMDS) to demonstrate the differences in species‐level taxa among various samples, we found that both the 16S rRNA and MAGs showed significant differences between different sample groups, except that the bottom sediment and slope sediment were very similar in the 16S rRNA data (Figure S1).

**FIGURE 2:**
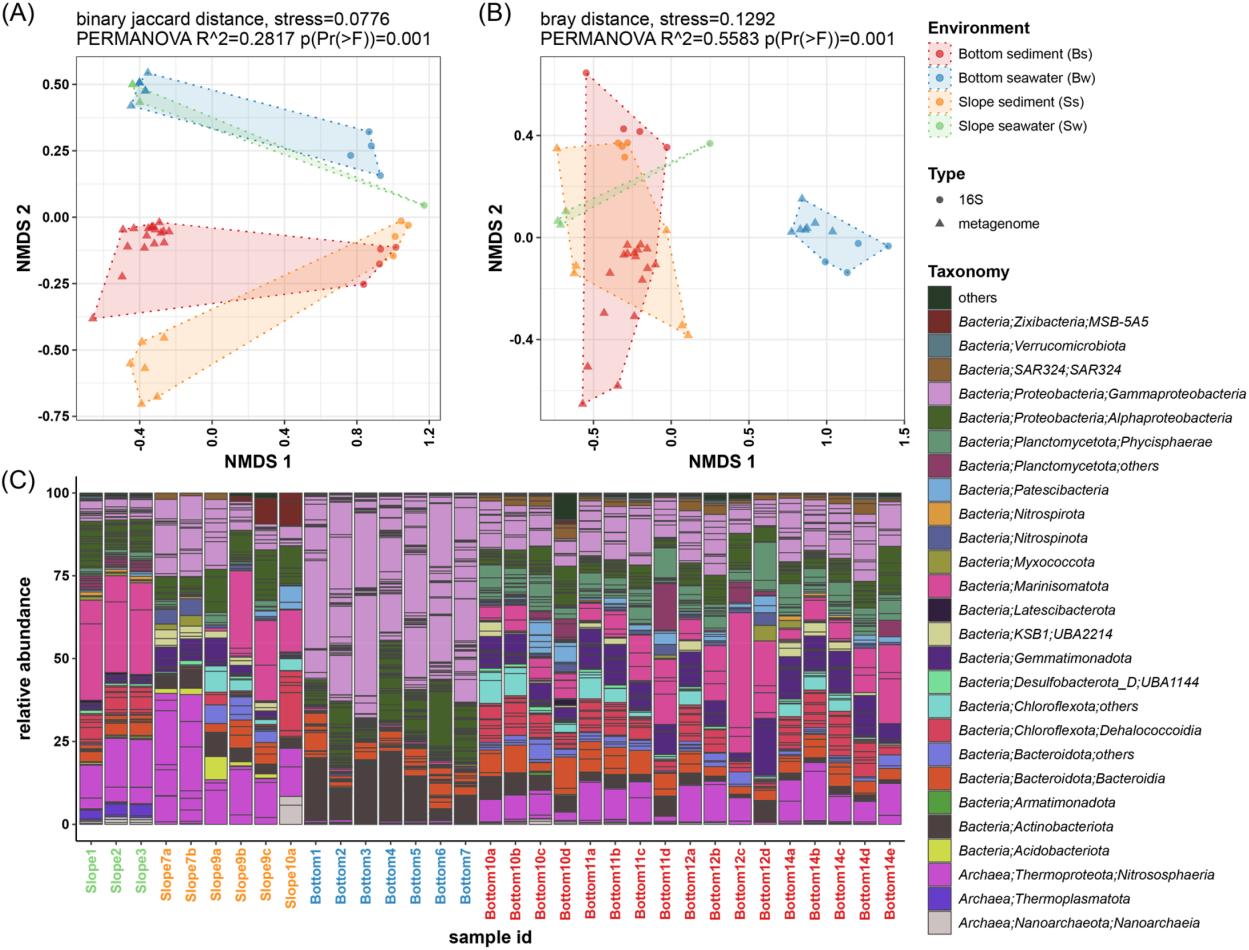
Taxonomy prevalence and abundance of classes in seawater and sediment samples at the slope and bottom of the Mariana Trench. (A) NMDS plot of binary Jaccard distance according to MAGs and 16S samples at the class level. (B) NMDS plot of Bray–Curtis distance according to MAGs and 16S samples at the class level. (C) Microbial community composition of all metagenome samples in this study. In (C), classes in the same phylum are coloured with the same colour, except for some specific abundant clades at the class level (e.g., *Alphaproteobacteria* and *Gammaproteobacteria* from *Proteobacteria*, *Dehalococcoidia* from *Chloroflexota*, and *Bacteroidia* from *Bacteroidota*) and the only classes of the phylum in this study (e.g., *UBA1144* from *Desulfobacterota_D*, *Nitrososphaeria* from *Thermoproteota*, and *Nanoarchaeia* from *Nanoarchaeota*).

To compare the community structure provided by both types of samples, we manually corrected the class‐level annotation of metagenomic and 16S rRNA data. In general, when considering the prevalence of taxa at the class level in the samples, samples were separated both by environment (seawater or sediment) and sequencing type (16S rRNA or metagenome) (Binary Jaccard distance, PERMANOVA test, *p* value <0.001) (Figure 2A). However, when considering the abundance of each class‐level taxon in the community among the samples of these four groups, the slope water samples were much closer to the sediment samples but were significantly separated from the bottom water samples (Bray–Curtis distance, PERMANOVA test, *p* value <0.001) (Figure 2B). Meanwhile, we did not observe significant differences caused by different filtration methods.

With respect to the taxonomic composition at the phylum level and at the class level for some specific clades, *Gammaproteobacteria* and *Alphaproteobacteria* were the most abundant bacterial taxa, especially in bottom seawater, and *Marinisomatia* was also abundant in some samples from the slope. *Nitrososphaeria* (previously reported as *Thaumarchaeota*, the typical ammonia‐oxidizing archaea, AOA) (Jung et al., 2022; Zhou et al., 2022) were the dominant archaea in all samples as well as an abundant taxon in most of the samples except for those from bottom seawater (Figure 2C, Figure S3).

**FIGURE 3:**
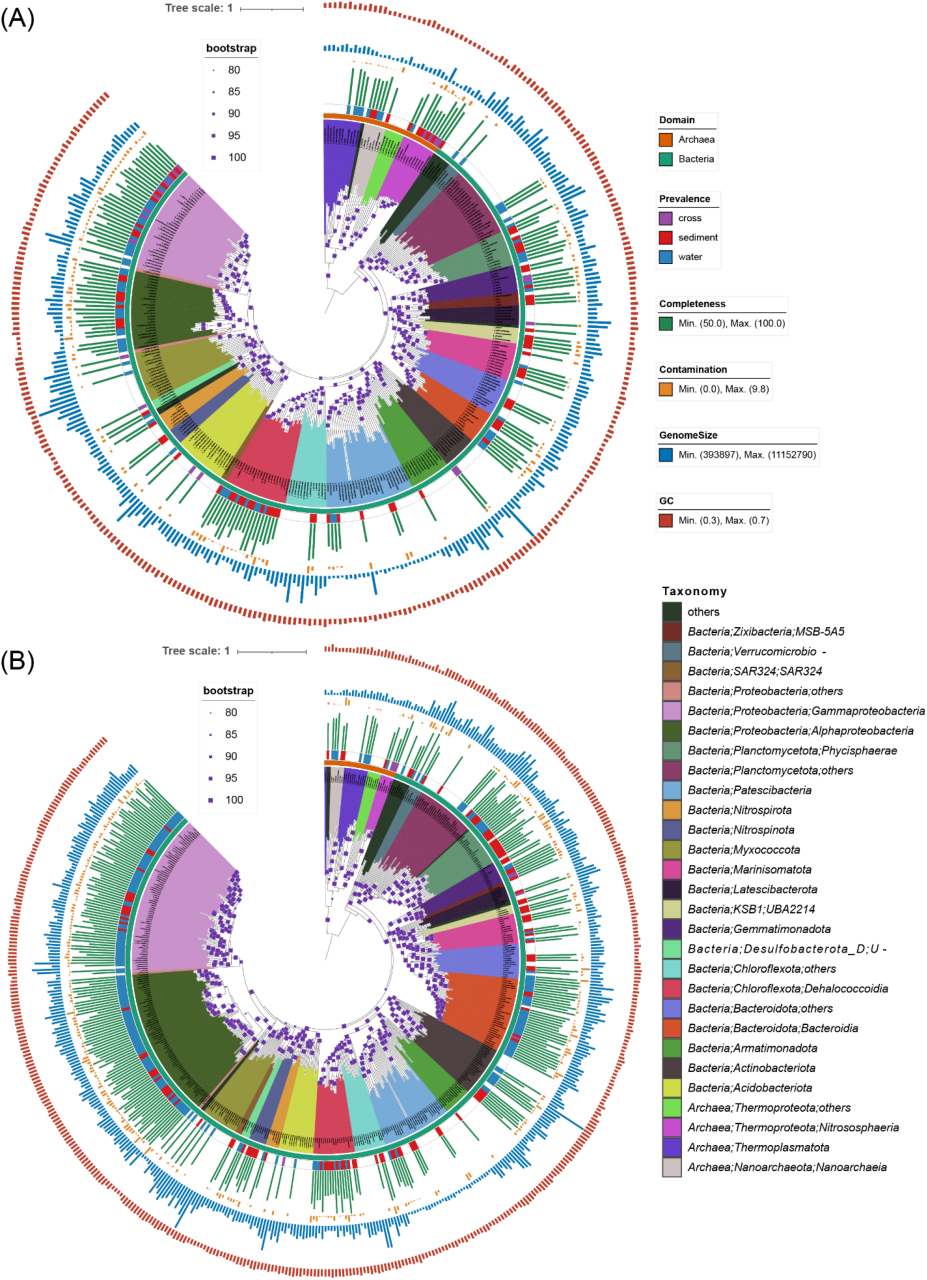
Phylogeny of metagenome‐assembled genomes (MAGs) from seawater and sediment samples from the bottom (A) and slope (B) in the Mariana Trench. Assembled genomes are dereplicated and annotated with GTDBTK (Chaumeil et al., 2019). Genomes that appeared only in sediment are marked with red, those that appeared only in seawater are marked with blue, and those that occurred across seawater and sediment at the bottom or slope are marked with pink.

We further constructed a phylogenetic tree by using 176 MAGs detected in the slope samples (slope seawater and slope sediment) and 323 MAGs detected in the bottom samples (bottom seawater and bottom sediment), together with 161 reference genomes from the Genome Taxonomy Database (GTDB) (Chaumeil et al., 2019) (Methods, Figure 3). These MAGs fell into 25 phyla in the slope samples and 28 phyla in the bottom samples (Figures S3 and S4). The class‐level clades across the seawater and sediment at the slope were more numerous and more widely distributed than those at the bottom of the Challenger Deep (Figures 3A,B and S4). While significantly higher alpha diversities were found in slope samples than bottom samples according to 16S rRNA data (*Wilcox* test, *p* value <0.05), the pattern of community composition and diversity was consistent with metagenomic data. A detailed comparison of the microbial communities in the seawater and sediment samples from the bottom and the southern slope will be described in the following section.

The microbial community composition in the seawater and sediment at the bottom of the Challenger Deep was completely distinct (Figures 2A,B, S1, and S3). In bottom seawater, *Gammaproteobacteria*, *Alphaproteobacteria*, *Actinomycetia*, and *Bacteroidota* were dominant, accounting for more than 94.7% of the total taxa, with *Gammaproteobacteria* being the most abundant class‐level clade, accounting for 44.4%–67.8% of the total in all seven bottom seawater samples (Figure 2C). However, the dominant class‐level taxa in the bottom sediment had significantly different proportions from those in the bottom seawater. Although *Gammaproteobacteria* was still among the dominant clades, its proportion was significantly lower, accounting for less than 24.4% of the total taxa in the communities of all bottom sediment samples (Figures 2C and S3; Table S2). In contrast, *Nitrososphaeria*, *Marinisomatota*, *Gemmatimonadota*, *Planctomycetota* (*Phycisphaerae* and others), and *Chloroflexota* (*Dehalococcoidia* and others) accounted for a notable proportion in the bottom sediment (up to ~66%), although their proportions were quite low among all samples in the bottom seawater (less than 3.16%).

In contrast to the finding at the bottom, the compositions in the slope seawater were more similar to those in the sediment on the slope (Figures 2A,B, and S3). Despite the heterogeneity among the slope sediment samples, the relative abundance of the dominant taxa at the class level was generally comparable between slope seawater and slope sediment. *Alphaproteobacteria*, *Marinisomatota*, and *Nitrososphaeria* were the three dominant class‐level clades in most slope seawater and slope sediment samples with very similar proportions (Figure 2C). However, the abundance of *Nitrososphaeria* in slope sediment was lower in the estimation from 16S rRNA data. In addition, the proportions of *Gammaproteobacteria* were also comparable between slope seawater and slope sediment on the southern slope, and they were much lower (22%) than those in the bottom sediment (Figure S3). In this way, the slope of the trench may act as a bridge for community exchange between seawater and sediment.

Both MAG‐based and 16S rRNA ASV‐based results consistently showed that the microbial members shared by both seawater and sediment samples constitute a significant part of the microbial communities (up to 30% based on MAG abundances and up to 70% based on ASV abundances, Figure S2). In addition, the interchange (indicated by the proportion of seawater‐obtained MAGs and ASVs) decreased in the deeper layer of the push core and reached the deepest layer involved in this study (46 cm in the slope and 21 cm in the bottom).

### Comparison of metabolic capabilities in nitrogen cycling between the seawater and sediment on the slope and bottom

**FIGURE 4:**
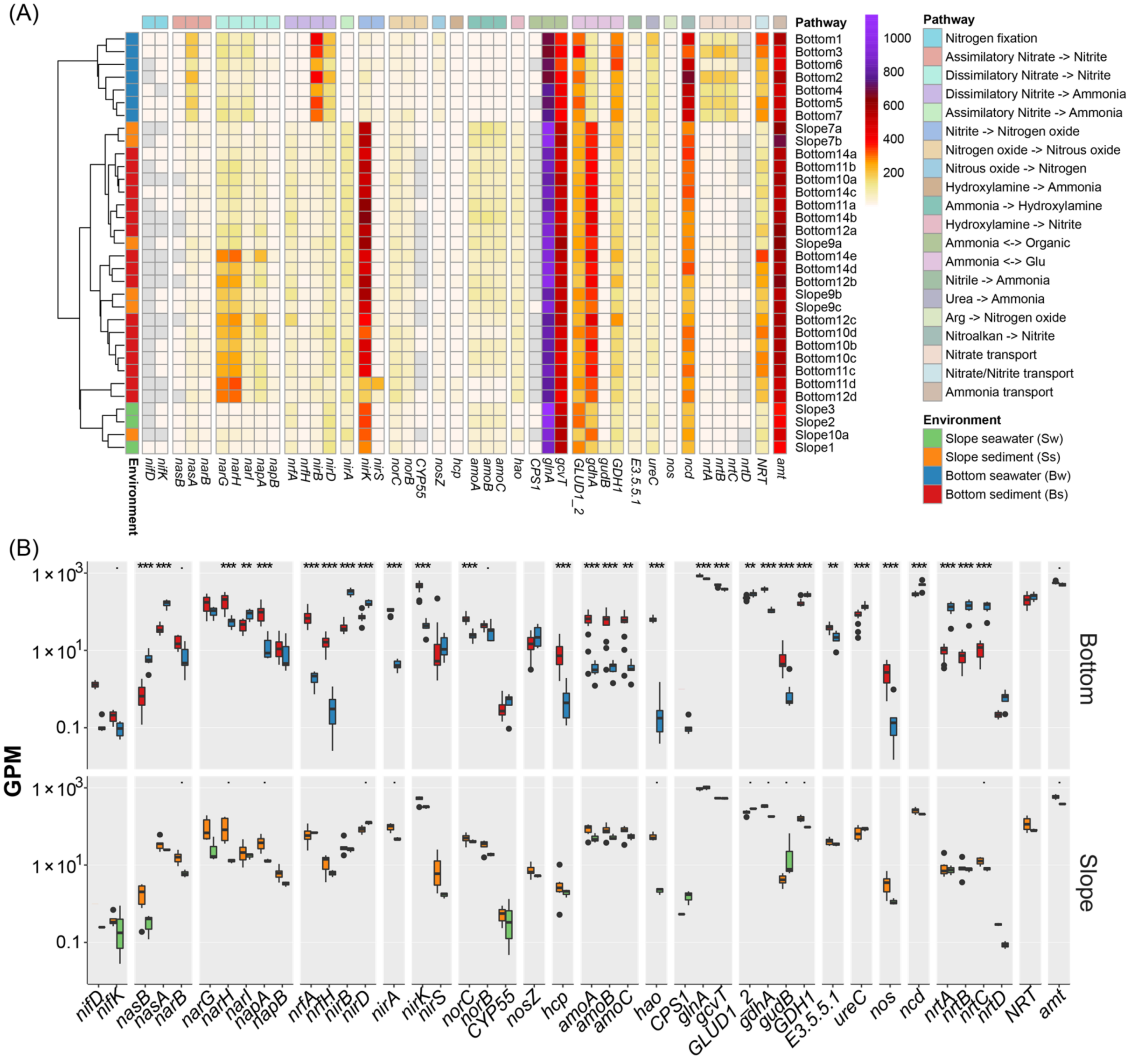
Relative abundance of metabolic genes involved in nitrogen metabolism from seawater and sediment samples collected at the slope and the bottom of the Mariana Trench. Gene abundance from different samples was estimated by the GPM method. (A) Total relative abundance of genes in different samples. (B) Average relative abundance and difference of genes in nitrogen metabolism in MAGs. The significance of differences between groups was examined by the *Wilcox* test; ‘.’, ‘*’, ‘**’, and ‘***’ indicate statistical significance at the FDR‐adjusted *p* < 0.1, 0.05, 0.01, and 0.001 levels, respectively.

**FIGURE 5:**
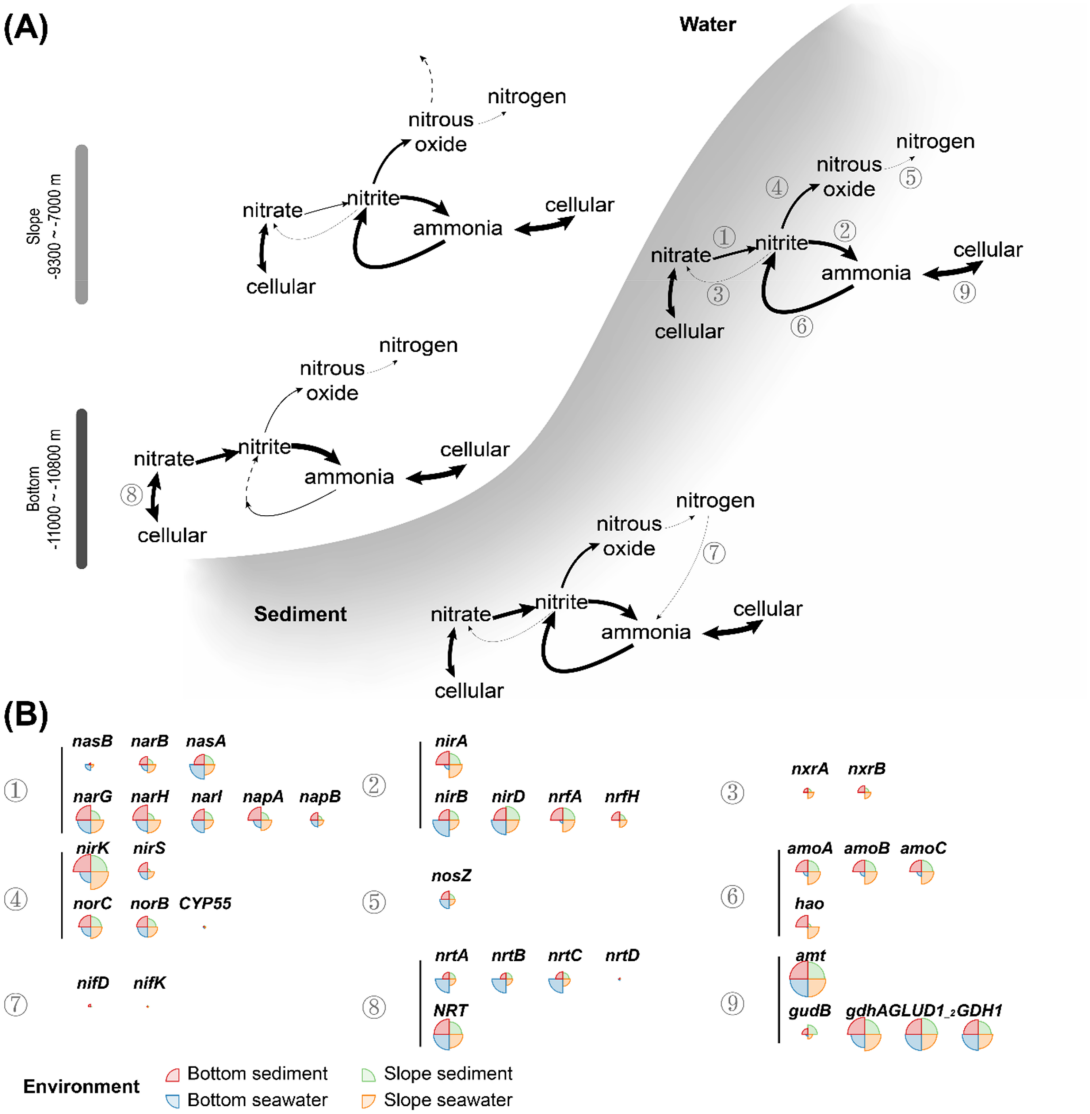
Schematic diagram of nitrogen cycling pathways in the seawater and sediments at the bottom and slope of the Challenger Deep. (A) The schematic diagram in slope seawater, slope sediment, bottom seawater, and bottom sediment. (B) The relative abundance of key metabolic genes. Detailed nitrogen‐related pathways: ① assimilatory and dissimilatory nitrate reduction; ② assimilatory and dissimilatory nitrite reduction; ③ nitrite oxidation; ④ nitrite reduction and nitrogen oxide reduction in denitrification; ⑤ nitrous oxide reduction; ⑥ ammonia oxidation; ⑦ nitrogen fixation; ⑧ nitrate transport; ⑨ ammonia transport and utilization. The thickness of the arrows indicates the potential capability (i.e., the relative abundance of genes) of the metabolic function, and the dashed arrows indicate the function/process for which no gene was detected but which is expected to occur.

Interestingly, the above taxa, which vary widely among different types of samples, were all associated with nitrogen cycling, suggesting potentially different capabilities in the nitrogen cycle between the seawater and sediment of the bottom and slope of the Challenger Deep. We further focused on the abundance of key metabolic genes from MAGs (Figures 4 and S5A–D). In general, key genes related to the nitrogen cycle were found to be widespread in the MAGs in all the measured seawater and sediment samples. Therefore, we used these key genes to indicate the various pathways related to the nitrogen cycle and highlighted the corresponding taxa using the key genes in the MAGs (Figures 4A and S6; Table S3). For the redox processes among major nitrogen species, the pathways of dissimilatory nitrate reduction to ammonia (*napA/nasA/narB* and *nirA/nirB/nrfA*) and denitrification (*nirK*, *norB* and *nosZ*) were relatively widely distributed within various class‐level clades, covering both the abovementioned abundant taxa (e.g., *Gammaproteobacteria*, *Alphaproteobacteria*, *Marinisomatia*, *Gemmatimonadetes*, *Planctomycetes*, *Dehalococcoidia*) and specific minor taxa (e.g., *Nitrospiria*). The representative gene of the system for the cleavage of organic nitrogen to form ammonium (*gcvT*, *GLUD1_2*, *gdhA*, and *npd*) and the transfer of nitrogen between amino acids and ammonia (*glnA*) and the ammonium transporter gene *amt* also had the widest distribution among all tested class‐level clades, which indicates the potential importance of the recycling and exchange of nitrogen sources between cells and inorganic ammonium in both the seawater and sediment on both the bottom and slope of the Challenger Deep (Figure S6). Genes related to ammonia (*amoABC*, *gcvT*, *GLUD1_2*, *gdhA*, and *npd*) were very abundant in all environments (Figure 5A,B). In contrast, some processes were constrained within certain clades, such as ammonia oxidation (*amoABC*) in *Nitrososphaeria* and nitrogen fixation (*nifD* and *nifK*) in one MAG from the genus *Bradyrhizobium* of *Alphaproteobacteria* (Figure S6). A comparison of the four groups of samples in terms of the relative abundance of nitrogen‐related genes revealed that the nitrogen cycling processes between the seawater and sediment on the slope and at the bottom were significantly different.

At the bottom of the trench, the abundance of many key nitrogen metabolic genes significantly differed between the seawater and sediment, with greater gene abundance for the DNRA (dissimilatory reduction of nitrate to ammonia) pathway. For example, genes in the nitrate reduction pathway were more abundant in both bottom sediment and bottom seawater samples, but they may have been from different gene families and microbial classes (Figures 4B and 5). The highest abundance of assimilatory nitrate reduction genes (*nasAB*) was detected in the bottom sediment samples compared to the other three groups, and these were mostly from *Gammaproteobacteria*. Genes catalysing the first step of nitrate reduction and denitrification (*narGHI*, *napAB*) were more abundant in both bottom samples than in the slope samples. However, in the sediment samples, *narGHI* and *napAB* from *Dehalococcoidia*, *Gemmatimonadetes*, *Phycisphaerae*, *Alphaproteobacteria*, and *Marinisomatota* were the most abundant, while those in bottom sediment samples may have been related to genes encoded by *Gammaproteobacteria* genomes.

The communities in bottom sediment and bottom seawater both exhibited highly abundant metabolic potentials for nitrite reduction but with distinct genes. The nitrite reduction pathways in seawater were conducted by the dissimilatory genes *nirBD*, dissimilatory genes *nrfAH* and *nirBD* and the assimilatory gene *nirA* in sediment. Most of the *nirBDs* annotated from bottom seawater were related to *Gammaproteobacteria*, while *nirD* in bottom sediment was mostly from *Marinisomatia*, *Dehalococcoidia*, *Alphaproteobacteria*, and *Nitrospinia*. The nitrite dissimilatory genes *nrfA* and *nrfH* showed constant co‐occurrence patterns across MAGs in the bottom seawater and bottom sediment samples (Figure S7A–C). However, the *Gammaproteobacteria* MAGs only contained *nrfA*, with no *nrfH* detected (Figure S6). The above results indicated that the utilisation of nitrite was conducted by different microbial clades using distinct enzymes between bottom sediment and bottom seawater in the Challenger Deep.

Compared to the bottom, higher gene abundance for the pathways of ammonia oxidation to nitrite was revealed on the slope. In general, the abundances of genes in slope sediment and slope seawater were more comparable than those in bottom sediment and bottom seawater samples. Genes involved in many pathways exhibited a similar abundance in the slope sediment and slope seawater samples as well as bottom sediment samples, including dissimilatory nitrate reduction (*narGHI*, *napAB*), assimilatory and dissimilatory nitrite reduction (*nrfAH*, *nirBD*, and *nirA*), ammonia oxidation (*amoABC*), and nitrate import (*nrtABC*). The high abundance of nitrite reduction and ammonia oxidation genes indicated the recycling of nitrogen between nitrite (reduced by bacteria) and ammonia (mainly oxidized by archaea) (Figure S6). In contrast, the abundance of *hcp*, which encodes an enzyme catalysing the decomposition of hydroxylamine to ammonia, was lower in slope sediment, slope seawater, and bottom seawater than in bottom sediment. We also noticed that while the total abundance of some genes was similar between slope sediment and slope seawater, they may be encoded by different genomes. For example, the most abundant *nrfA* gene was encoded by *Marinisomatia* in slope seawater and *Ignavibacteria* from the *Bacteroidota* in slope sediment. In addition, a high abundance of *nirK* from *Nitrososphaeria* and several bacterial lineages was detected in both the slope sediment and slope seawater samples, while another denitrification gene, *nirS*, was mostly encoded by *Marinisomatia* in the slope sediment samples and nearly absent in the slope seawater samples.

Interestingly, niche differentiation of *Nitrososphaeria* was obtained in slope sediment and slope seawater of the Mariana Trench. A total of seven MAG of *Nitrososphaeria* in this study were detected with *amo* genes, including three MAGs found in slope sediment only, two detected in all environments, one slope only and one in seawater only (Figure S8A,B; Table S4 **)**. The only AOA MAG in the seawater was annotated as family *Nitrosopelagicus* and was in a different clade compared to all other AOA MAGs that could be found in sediment samples. The only AOA MAG on the slope present in both seawater and sediment samples was annotated as belonging to the placeholder genus JACEMX01 of the family *Nitrosopumilaceae*. While *gdhA*, *nirK*, *glnA*, *amt* and *amoAB* were annotated from the JACEMX01 MAG, the abundance of its *gdhA* in a slope seawater sample is 2–3 times higher than that in slope sediment samples, and another gene (*ubiE* in ubiquinone biosynthesis) on the same contig of *gdhA* was also detected with higher abundance in the slope seawater sample. Both DRGT01 MAGs were only found in slope sediment, which has been reported to be dominant in the abyssal sediment (4000–6000 m b.s.l) in Kermadec Trench and Atacama Trench (Trouche et al., 2023). The remaining two widespread AOA MAGs, together with a slope seawater‐only MAG were annotated as belonging to the genus *Nitrosopumilus*, including one of the most abundant MAGs (cluster 82_2, represented by TY.041‐concoct_51) in bottom sediment and slope seawater. Cluster 82_2 was annotated as Ca. *Nitrosopumilus* sp. *MTA1* (Zhong et al., 2020), including 11 redundant metagenomic bins from bottom seawater, slope seawater, and one bottom sediment sample. Compared to bins recovered from seawater samples, several genes were missed in the bin origin from sediment, including *speG* in GABA biosynthesis, and *asrCR* for arsenate resistance. Compared to the high abundance of MAG and other genes, the relative abundances of these genes were significantly lower.

### Comparison of metabolic capabilities in sulphur cycling between the seawater and sediment on the slope and bottom

We next focused on key metabolic genes related to the sulphur cycle and found that the abundance of sulphur metabolism genes in bottom seawater was generally lower than that in the other groups. The abundances of enzymes for dissimilatory sulphate reduction (*Sat*, *aprAB*) and assimilatory sulphite reduction (*sir*) were significantly lower in bottom seawater than in the other three groups. In the bottom seawater samples, a higher abundance of assimilatory enzymes (*cysN*, *cysD*, and *cysJ*) was observed, and most *cysJ* genes were carried by *Gammaproteobacteria* MAGs. Other genes involved in the assimilatory pathway (*cysC*, *cysH*, and *cysI*) were commonly used in all four groups.

The enzyme for assimilatory sulphide utilisation (*cysK*) was widely distributed in diverse lineages. Interestingly, while the dominant *cysK* genes in bottom sediment, slope sediment, and slope seawater were annotated from *Nitrososphaeria* MAGs, it was annotated as *Gammaproteobacteria* in bottom seawater. In addition, the high potential of sulphur oxidization was indicated by a high abundance of gene encoding sulphide: quinone oxidoreductase (*sqr*). Genes encoding the SOX complex for thiosulphate oxidation were also annotated from all four sample groups. The abundance of all SOX genes was significantly lower in bottom seawater than in the other three groups. In seawater samples, thiosulphate was also likely to be catalysed to sulphite given the higher abundance of *glpE* compared to that in sediment samples from both the bottom and slope.

### Dynamic processes of the microbial community on the slope and bottom

**FIGURE 6:**
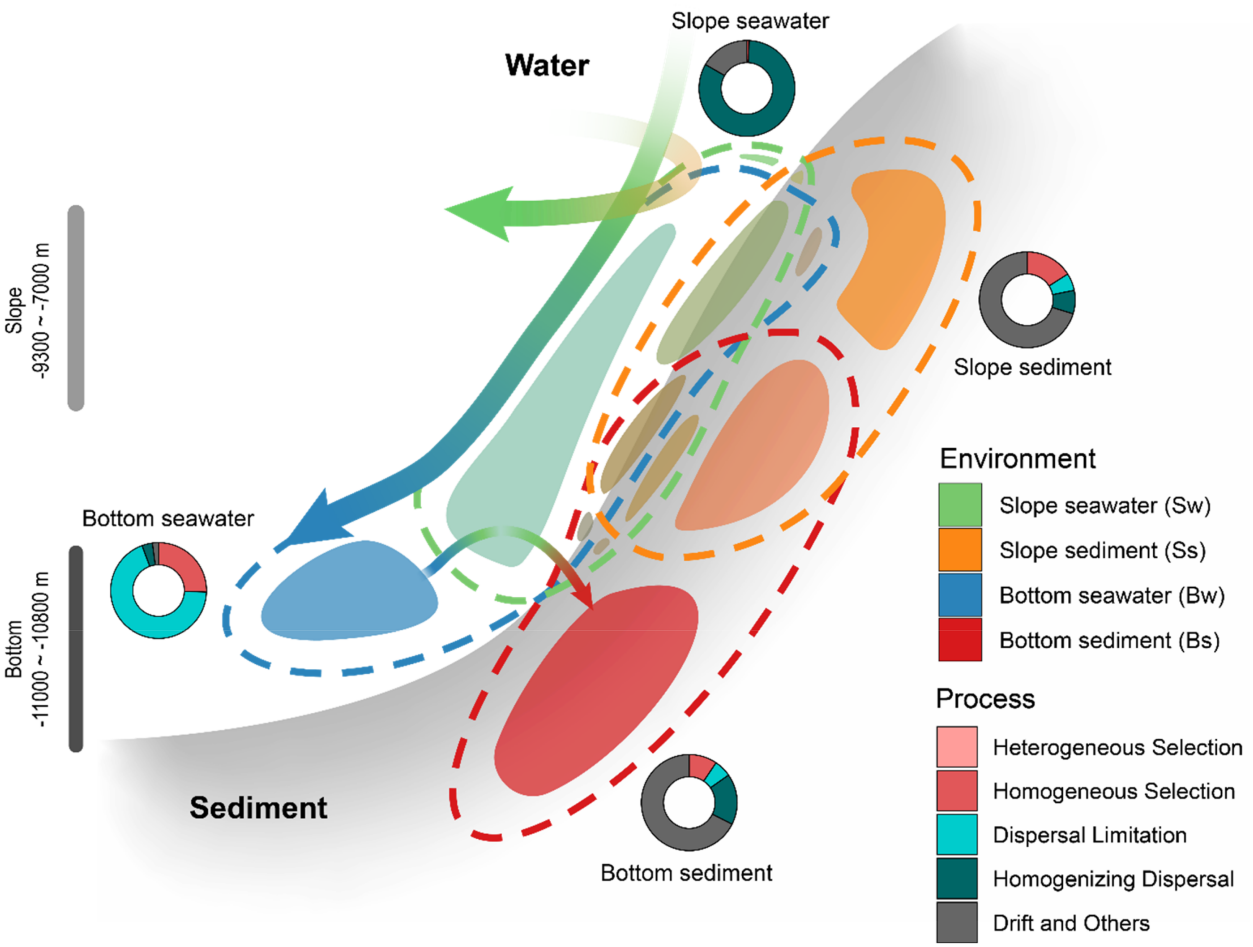
Schematic diagram of the taxonomic composition of the microbial community in the slope and bottom of the Mariana Trench. The dashed line indicates genome sets detected in different environments, and the colour blocks indicate the size of the subset shared across environments according to Figure S3. The ring plot shows the driving force shaping the community in the environment. The two wider arrows indicate the direction of estimated water flux, and the thinner arrow indicates the estimated net microbial population movement.

To assess the driving forces underlying the observed differences in microbial composition and element cycling potentials in the seawater and sediment of the bottom and slope, we calculated the relative contributions of selective (heterogeneous selection and homogeneous selection) and stochastic (dispersal limitation, homogenizing dispersal, drift and others) processes to the microbial community assemblage (Figure S9). Stochastic processes dominate the formation of microbial communities in the Challenger Deep. The seawater communities in the slope and bottom samples were mostly shaped by homogenizing dispersal (82.6%) and dispersal limitation (68.9%), respectively, while sediment communities were mainly driven by drift and other processes (70.2% and 69.6% in the bottom and slope, respectively) (Figure 6). Notably, the contribution of the dispersal limitation process, which represents the difficulty of exchange between microbial communities, was lower between the slope seawater and slope sediment groups (37.8%) than between the bottom seawater and bottom sediment groups (42.5%). Compared to slope sediment vs. slope seawater, relatively higher contribution of dispersal limitation (42%) and lower for homogeneous selection (6%) were observed in bottom sediment vs. bottom seawater (38% and 12%, respectively). Generally, consistent trends were observed between the comparison of community assembly based on MAG and 16S rRNA calculations (Figure S9).

We further estimated the net population movement between samples based on the abundance distributions and origins of MAGs. The pairwise interactions between samples were classified into four groups, including transmitted (significant movement in one direction), mixed (significant bidirectional movement), isolated (limited movement compared to normal) and others (Methods). Significant movement was detected from bottom seawater samples to slope seawater samples (average net flux, 43 RPKM) and slope seawater samples to sediment samples (average net flux, 27 RPKM) (Figure 6). Interactions between bottom seawater and bottom sediment samples and between bottom sediment and slope sediment samples were identified as either isolated or others. Significant pairwise mixing was observed between some of the samples within the bottom seawater group (Figure S10).

## DISCUSSION

The Challenger Deep of the Mariana Trench is the deepest region of the ocean. Its water depth of up to 11 km b.s.l. and its V‐shaped topography jointly result in different habitats between the bottom and the slope and impact the ocean circulation structure (Jiang et al., 2021; Zhou et al., 2022). However, the HHP at a water depth of 11 km in the Challenger Deep poses a technical challenge for in situ sampling and measurements. By comparing the microbial communities at the bottom and on the slope of the Challenger Deep between the near‐benthic seawater and the sediment, we observed that distinct niche differentiation may be caused by seawater‐sediment interaction processes at different topographical locations within the Challenger Deep (Figures 2, 3 and 5). This result indicated a possible occurrence of more pronounced seawater‐sediment deposition and interchange on the slope (7–9 km), which could be both in processing and presented in the past, and more significant geographic isolation at the bottom (9–11 km) of the Challenger Deep, which is likely facilitating the formation of diffusion restrictions and niche separation. Meanwhile, metagenomics‐based community assembly analyses also revealed stronger homogeneous dispersal in the seawater on the slope and more limited dispersal at the bottom. Seawater and sediment samples were collected in adjacent years and the distance between slope samples is greater than that between bottom samples. Therefore, the observed phenomenon, that is, a high level of similarity between the taxa of sediment and water along the slope, is widespread across the area. This suggests that the frequent sediment resuspension observed on the slope is a property of the environment itself rather than a result of sampling. While all the seawater samples we collected using Lander (Methods), those from the trench bottom still show significant differences from the sediment, further confirming the stability of our conclusions. All these results, from different aspects, indicated that the ecological niche differentiation of microbial community composition might be influenced by marine geological conditions and hydrological factors, which is related to the availability of organic matter and redox pairs, and/or stochastic processes such as currents (Chen et al., 2023; Jing et al., 2022; Yap et al., 2021; Zhou et al., 2022). The trench sediment ecosystems were reported to be dominated by stochasticity in previous study (Sun et al., 2024), indicating that factors such as drift and dispersal are more likely to form and exist in trenches. In addition, the population movement analysis results indicate that exchange processes occur between microbial communities on the slope, both within the seawater and between the seawater and the sediment.

Furthermore, the results here also demonstrated the differentiation of metabolic potential involved in elemental cycling (Figures 4 and 5). Using nitrogen cycling processes as an example, the abundance of key enzymes for different nitrogen cycling pathways in the metagenomes indicated the distinct metabolic capacities and preferences of the communities in the bottom and slope environments. Similar to nitrogen cycling, abundant and distinct sulphate and sulphite reduction genes were observed between the slope and bottom. Nitrate and sulphate are common electron acceptors that follow oxygen, and their reduction was reported to be enhanced under high pressure (Li et al., 2018). Compared with aerobic respiration, anaerobic respiration processes, such as nitrate reduction, may reduce the production of ROS, and microbes may prefer nitrate as an electronic accepter instead of O_2_ under HHP conditions. This may explain the high metabolic potential of anaerobic respiration in aerobic seawater and surface sediment with oxygen concentration more than 50 μM (Zhou et al., 2022). In addition, the stronger nitrate reduction potentials at the bottom of the Challenger Deep could provide extra energy for organic matter degradation beyond aerobic respiration. A higher abundance of organic carbon degradation genes, such as aromatic compounds (*catAE*, *boxB*, and *hcrA*) and complex sugars (*chi* and *pulA*), was observed at the bottom of the Challenger Deep (Supplemental Results, Figure S4A–I). However, on the slope, higher recycling of ammonia oxidation to nitrite than at the bottom was observed, which was conducted by similar taxa using similar pathways and genes between the seawater and sediment. Ammonia could be released during the degradation of organic matter, and there may be a trophic cascade between the process of oxidative recirculation of ammonia and the degradation of organic matter (Jing et al., 2022). In addition, ammonia oxidation is also considered a major contributor to dark carbon fixation in the ocean, which may be involved in the regeneration of organic carbon in the Challenger Deep. The evidence of niche separation of AOA has also been detected among different samples. Various AOA clades are identified throughout the column of marine and consistently reported as one of the most important taxa in the hadal trench previously (Nunoura et al., 2015; Wang et al., 2019; Zhong et al., 2020). In this study, MAGs of AOA also emerged as dominant species and played primary roles in the initial step of nitrification in the sediments in both the bottom and slope zones of Challenger Deep. Notably, specialists (found only in Ss, seawater or slope samples) and generalist MAGs of AOA annotated as *Nitrososphaeria* were detected in this study. Even within the generalist *Nitrososphaeria* MAGs, different arsine resistant strategy was also detected, which is likely the consequence of ecological functional differentiation for niche adaptation from seawater to sediment.

Finally, our inference of the water movement from the slope is generally consistent with previous results obtained through in situ observations and calculations at a water depth of 7 km in the Challenger Deep (Jiang et al., 2021), which indicated the possibility of using metagenomic analysis to provide additional clues on oceanographic processes. The inferences here remain to be validated in larger‐scale sampling and more systematic investigations of the Challenger Deep in the Mariana Trench. Therefore, the difference in nitrogen cycling processes between the bottom and the slope and the seawater and the sediment is accompanied by carbon conversion, which is greatly important for a deeper understanding of elemental turnover in the Mariana Trench. The different abundance of key genomes and genes between environments also reflect the niche differentiation after dispersal and community exchange. This study provided some phenomenon of niche separation according to metagenomic information on the abundance of key genes, and more direct evidence obtained by integrating additional methodologies such as the metaproteomic approach and/or in situ measurements is still needed in the future.

## AUTHOR CONTRIBUTIONS

**Aoran Hu:** Methodology; formal analysis; writing – review and editing. **Weishu Zhao:** Writing – review and editing; writing – original draft; conceptualization; data curation. **Jing Wang:** Methodology; formal analysis. **Qi Qi:** Formal analysis; investigation. **Xiang Xiao:** Conceptualization; supervision. **Hongmei Jing:** Conceptualization; project administration; funding acquisition; writing – review and editing; resources.

## CONFLICT OF INTEREST STATEMENT

The authors declare no conflicts of interest.

## Supporting information


**Data S1.** Supplementary Information.

**Table S2.** Relative abundance of class‐level clades in samples from bottom seawater, bottom sediments, slope seawater and slope sediments.

**Table S3.** Representative gene list and relative abundancy of genes in N metabolism among all clean reads in different samples.

## Data Availability

The metagenomic datasets generated and analysed during the current study are available in the NCBI repository under the accession codes PRJNA859662 (for seawater) and PRJNA635214 (for sediments). The 16S rRNA datasets generated and analysed during the current study are available in the NCBI repository under the accession codes PRJNA1018490 (for seawater) and PRJNA854746 (for sediments). All original scripts and result tables to generate final figures are available in the GitHub repository: https://github.com/weishuzhao/MT-SwBw.
