# Supplementary material for: Microbial communities reveal niche partitioning across the slope and bottom zones of the challenger deep
Source: Environ Microbiol Rep. 2024 Jul 31;16(4):e13314. doi: 10.1111/1758-2229.13314 (PMC11291871; doi:10.1111/1758-2229.13314)
Supplement: Supplementary file 1 — Data S1. Supplementary Information. [file EMI4-16-e13314-s002.docx]

Supplemental Materials

# Supplemental Results

## Metabolic capabilities

### Carbon fixation

Key genes of 3PH-4HB pathway (*3hpcs*, *4hbcl*) were only obtained in *Nitrososphaeria*, which was widespread across all samples in the CD, and the abundance of these genes were relatively low in the bottom water. Key genes of rTCA (*aclA*, *ccsA*) were mostly found in *Nitrospinia* of *Nitrospinota*, while *aclA* was only find in *Nitrospiria* in water environments. This indicated the important role of carbon fixation of *Nitrospinia* in the sediment, and the intercellular cooperation for rTCA cycling in the water. On the contrary, key genes in CBB pathway (*prkB*) was abundant, and shared in various strains, mainly in *Gammaproteobacteria*.

### Complex carbon degergation.

For alkane degradation, *alkM* was the most abundant alkane monooxygenase in bottom and sediment water, while *ladA* was also abundant. Gens annotated as *alkM* had been found in various strain in *Proteobacteria*, *Myxococcota*. *alkM* was also found in *Poseidoniales* in *Thermoplasmatota*, which was mostly found in only bottom water. However, genomes containing *ladA* could be found in both bottom and slope water, and was more abundant in slope water.

Archaea in this study was obtained the potential to utilize aromatic components. Four *Poseidoniales* encoding genes *boxB* and *hcrA* could have the potential to degrade benzoyl. All these *Poseidoniales* could be found in water. However, *boxB* and *hcrA* were more abundant in sediment and slope water samples. Several gene, such as *catA* and *catC*, were found to be widespread in Bacteria and Archaea genomes, and had high abundance in every environment. However, *catA* was more abundant in water samples, while *catC* had much higher abundance in sediment samples followed by slope water samples. *badH* and *benA* were more abundant only in bottom, indicating a functional differentiation and nutrient difference between environment.

Key genes of complex sugar were more abundant in water, espcially *chi* and *pulA*, which was more abundant in slope and bottom water, respectively.

### Nitrogen metabolism

**Nitrogen fixation**: Nitrogen fixation genes had been found in multiple trenches except Mariana trench (zhang_2018, zhou_2022). In this study, *nifH* had not been detected in any represantative MAGs, but in a mid-quality MAG annotated as *Alteriqipengyuania* of *Sphingomonadales*, *Alphaproteobacteria*. *Sphingomonadales* was reported to have the potential to fix nitrogen. *nifDK* were found in a manual curated MAG assembled from the slope water samples, which is annotated as *Bradyrhizobiom*. However, the relative abundance of *nifDK* was highest in the bottom sediment, and unexpectedly undetected in slope sediment samples. This indicates that some unknown species and enzymes involved in nitrogen fixation may exist in sediment samples.

**Ammonia Oxidation**: *amoABC* were dectected in most environment and mostly annotated from *Nitrososphaeria* MAGs. Interestingly, a MAG annotated as *'Nitrosopumilus* *sp013390905'* have been found in all environments, and being the most abundant ammonia oxidizer in bottem sediments, bottom water and slope water. However, a MAG annotated to genus *'DRGT01'* are the dominant ammonia oxidizer in slope sediment. *Nitrosopumilus sp013390905* is also the main ammonia oxidizer in bottom water, while the relative abundance of *amo* genes was pretty low compared to other environments. A few *Gammaproteobacteria* genomes found in bottom and slope water also encoded *amo* genes, including *Immundisolibacter*, *Polycyclovorans*, *Ga0077536* and *Nitrosomonadaceae*. However, probably related to low MAG quality, the *Nitrosomonadaceae amo* operon was only found in the replicated MAG. The relative abundance of bacterial *hao* is relatively high in the sediment, and can be found in various genomes, mainly *Gemmatimonadetes* and *Marinisomatales*. No *hox* was detected in this study.

**Ammonia uptake / product**: Genes taking ammonia as substrate to synthesize organic matter or decomposing organic matter to produce ammonia were widely distributed and abundant in all samples and various classes. The relative abundance of *glnA* and *gcvT* in slope was higher than that in bottom environment. *CPS1*, which is only found in *Nitrospirales*, was low abundant in CD. The gene involved in urea reduction and nitrile reduction were also widespread. However, while *ureC* was more abundant in water, the relative abundance of gene *E3.5.5.1* was higher in the sediment. MAGs from *Alphaproteobacteria* had the potential to degrade *ureC* in all environments. The *ureC* from *Gammaproteobacteria* and *Actinomycetia* was more abundant in the bottom water, while *Nitrososphaeria* was the most important urea degrader in other samples.

**Nitrogen species transport**: Ammonia transporter amt could be found in various classes. *nrtABC* were only abundant found in the bottom water, and most of these genes were annotated from *Gammaproteobacteria*, which was also abundant in these environments. The nrt annoated from *Alteromonas macleodii* and *Halomonas aquamarina* were highest in bottom water. However, the relative abundance of *nrtD* was much lower and only found in a MAG annotated as *Lentisphaeria*.

# Supplemental Figures

## Figure. S1. Community composition and beta-diversity of different environment groups according to 16S rRNA gene and MAG abundance.

(A-X) Beta diversity of community belonging to the group of slope seawater (Sw), slope sediments (Ss), bottom seawater (Bs) and bottom sediments (Bs) described by 16S rRNA gene or MAG abundance at all phylogenetic status. (Y) Community composition according to 16S data and colored at order level.


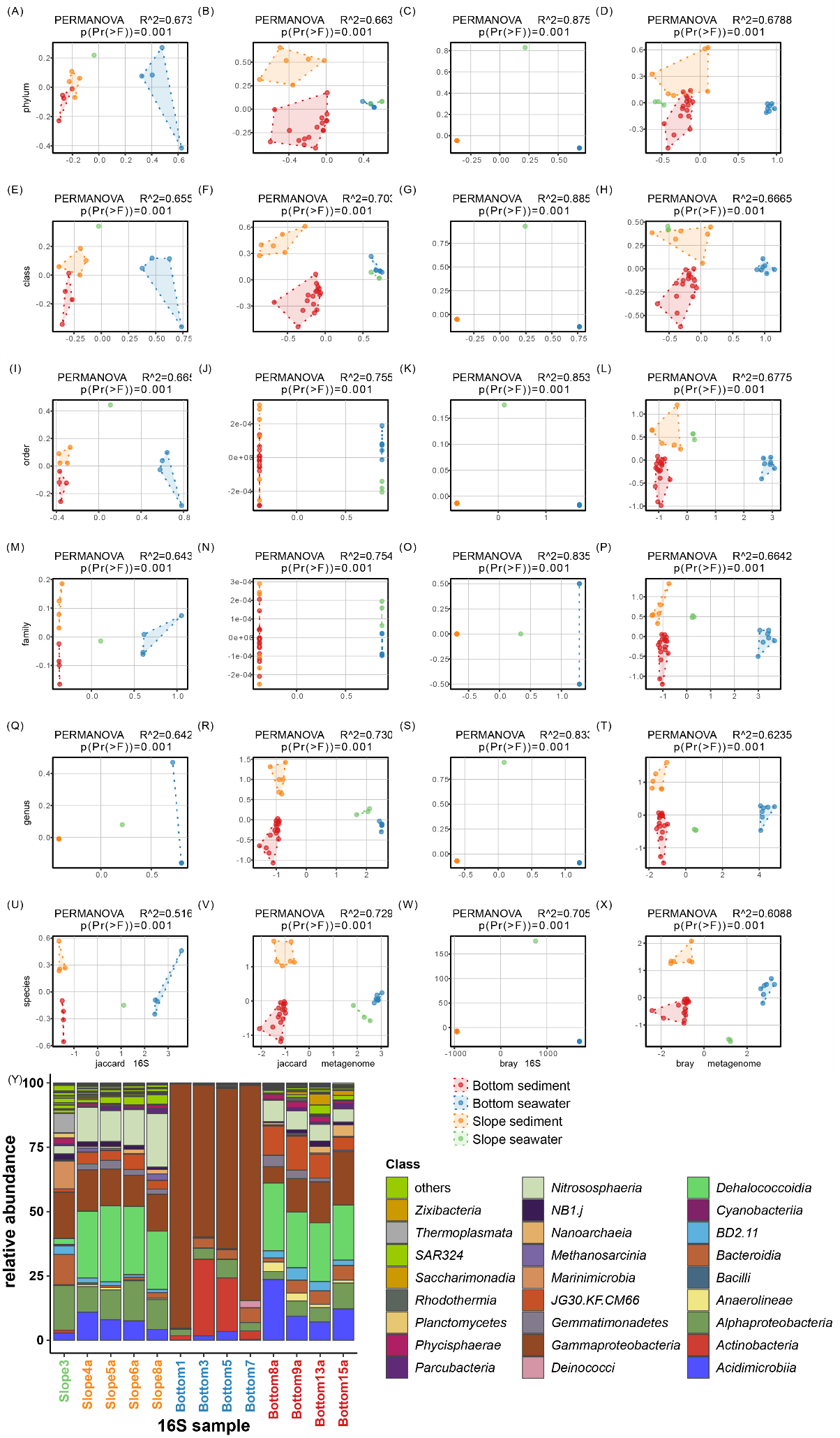


## Figure. S2. Exchange of microbial communities between sea-water and sediment.

The abundance of water only-strains (defined as MAGs/ASVs only found in water samples), sediment only-strains (only found in sediment samples) or share-strains (found in at least one water sample and at least one sediment sample).


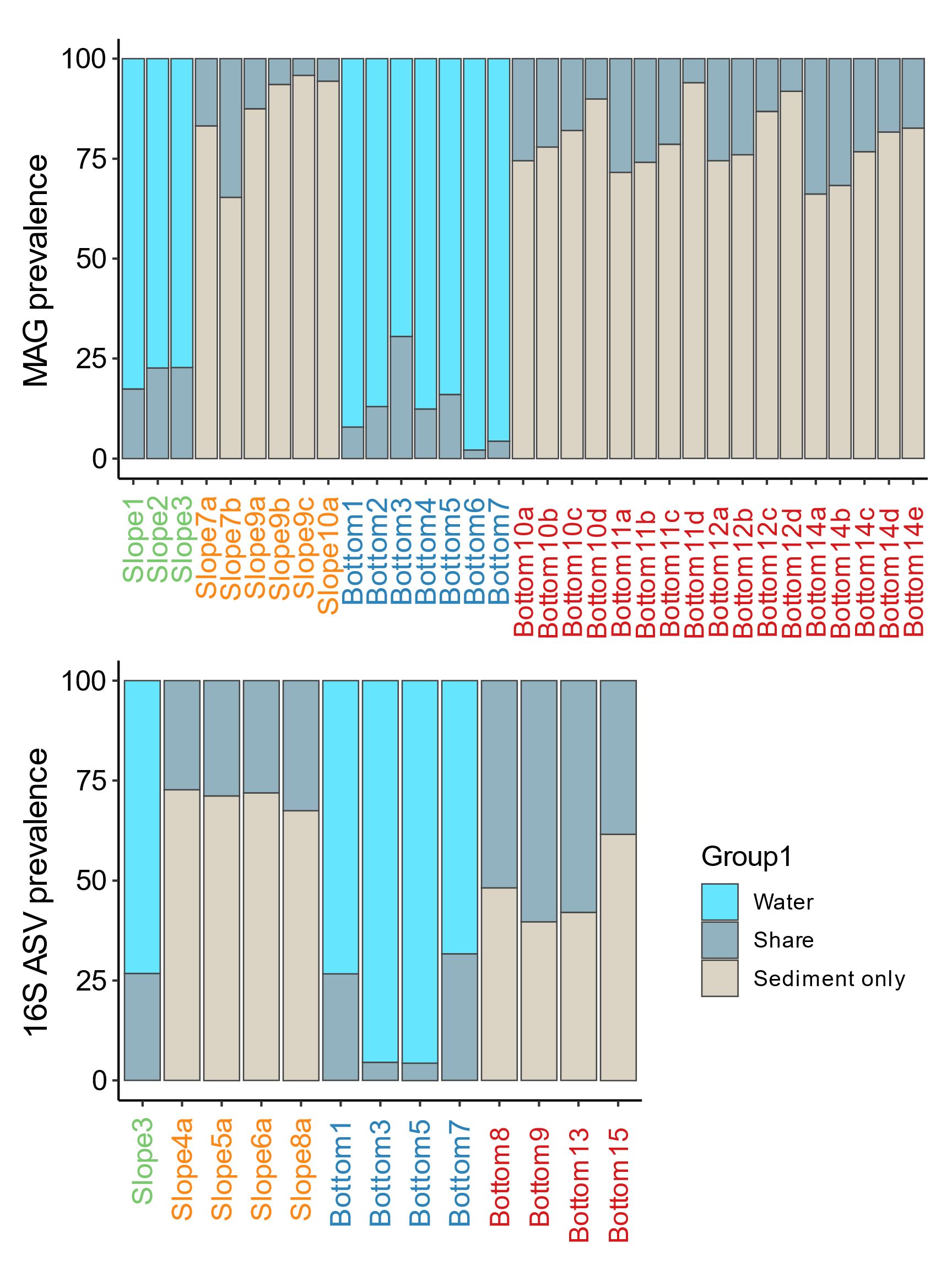


## Figure. S3. Relative abundance of all MAGs in different groups of samples.

Relative abundance of all MAGs belonging to the group of slope seawater (Sw), slope sediments (Ss), bottom seawater (Bs) and bottom sediments (Bs). Significant difference is calculated by Wilcox text in R and marked with “*”.

**
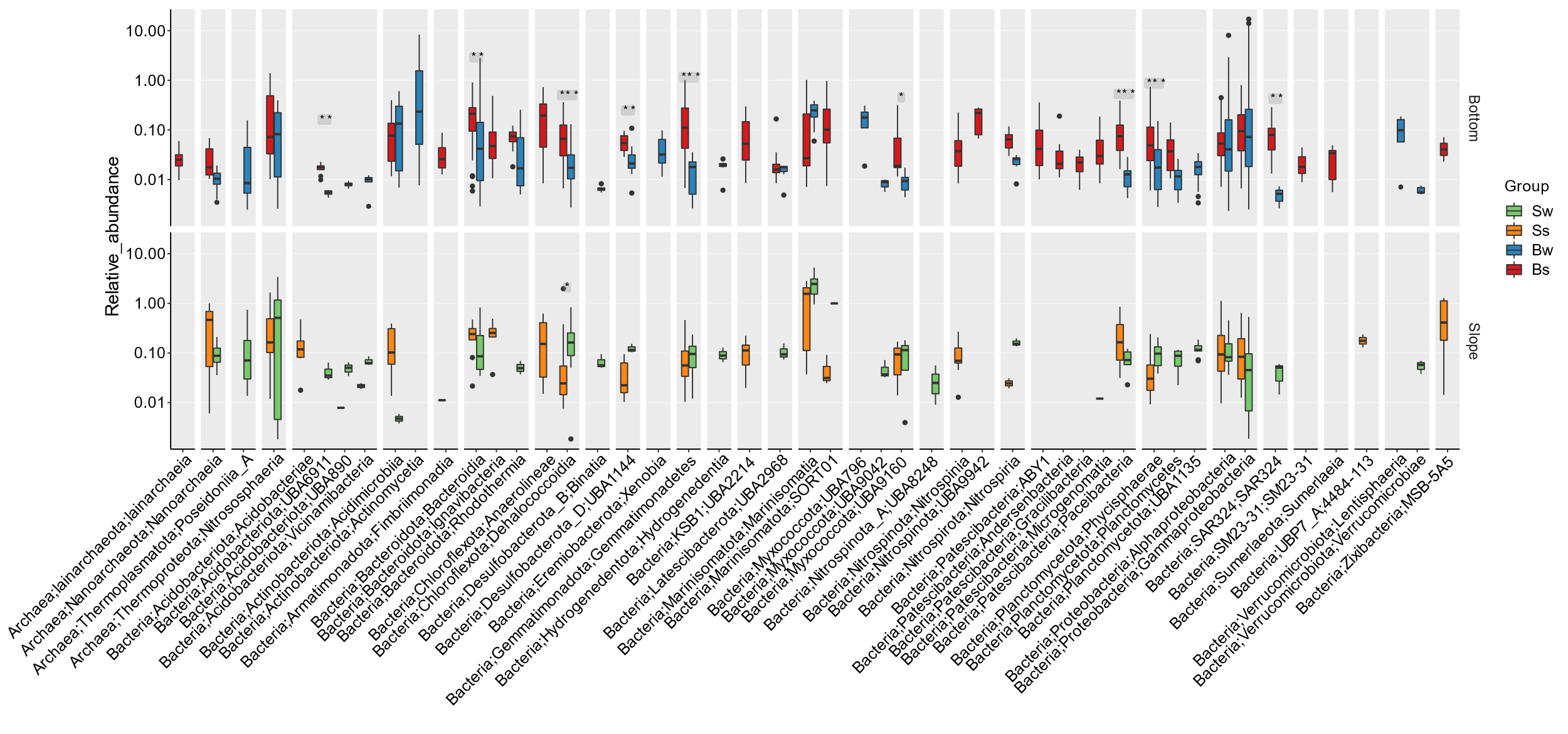
**

## Figure. S4. Venn plots of MAGs compared among different groups of samples.

Venn plots of MAGs at each taxonomic level (from domain to species), KOs and complete metabolic modules (> 50%) in slope seawater (Sw), slope sediments (Ss), bottom seawater (Bs) and bottom sediments (Bs).

**
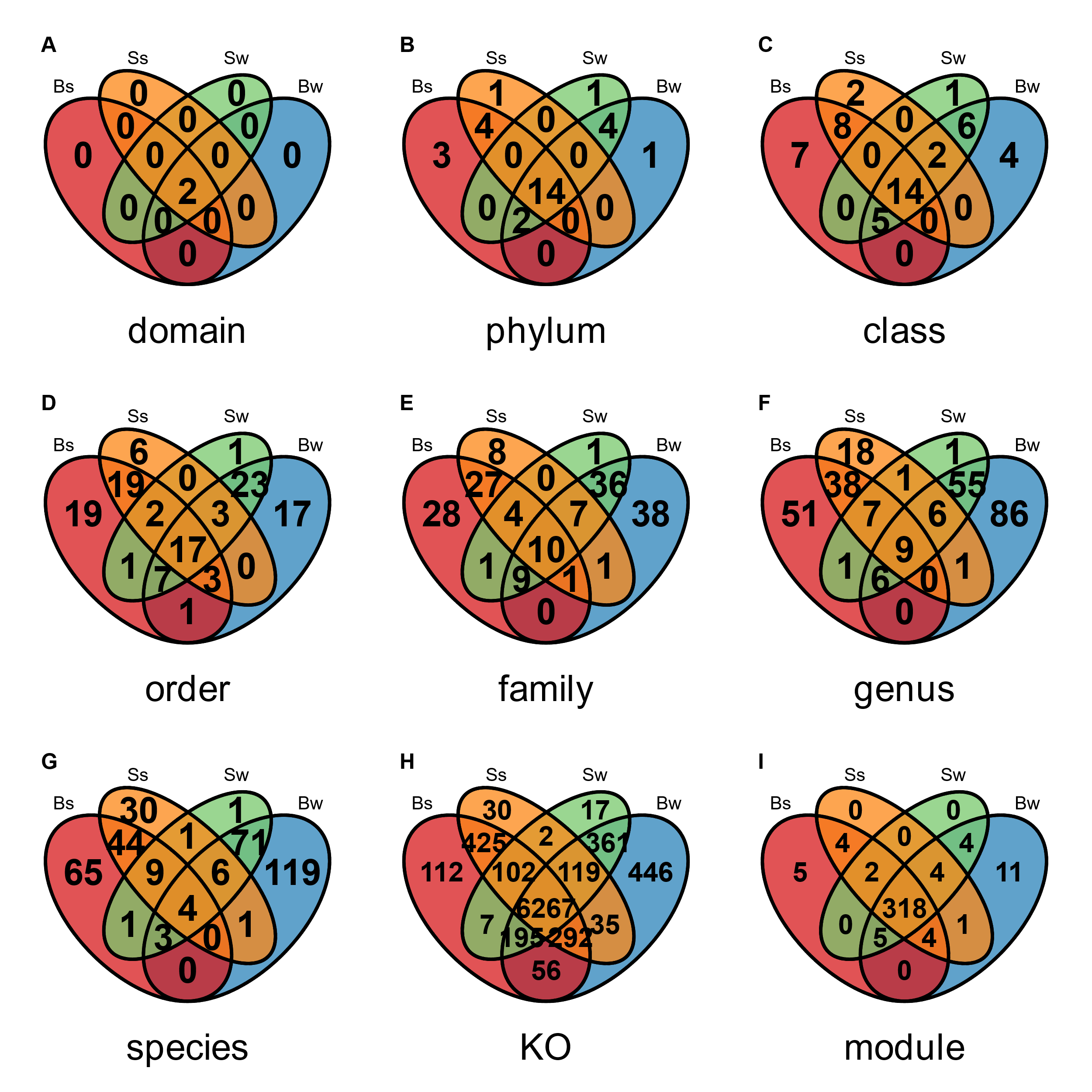
**

## Figure. S5. Relative abundance of representative metabolic genes in different groups of samples.

GPM of key metabolic genes for (A) CO2 fixation pathways and degradation of ROMs, (B) abiotic enantiomers metabolism, (C) complex sugars and hydrocarbons degradation, and (D) others between slope seawater (Sw), slope sediments (Ss), bottom seawater (Bs) and bottom sediments (Bs). Genes are corresponding to the gene IDs in Supplemental Table S2.

**
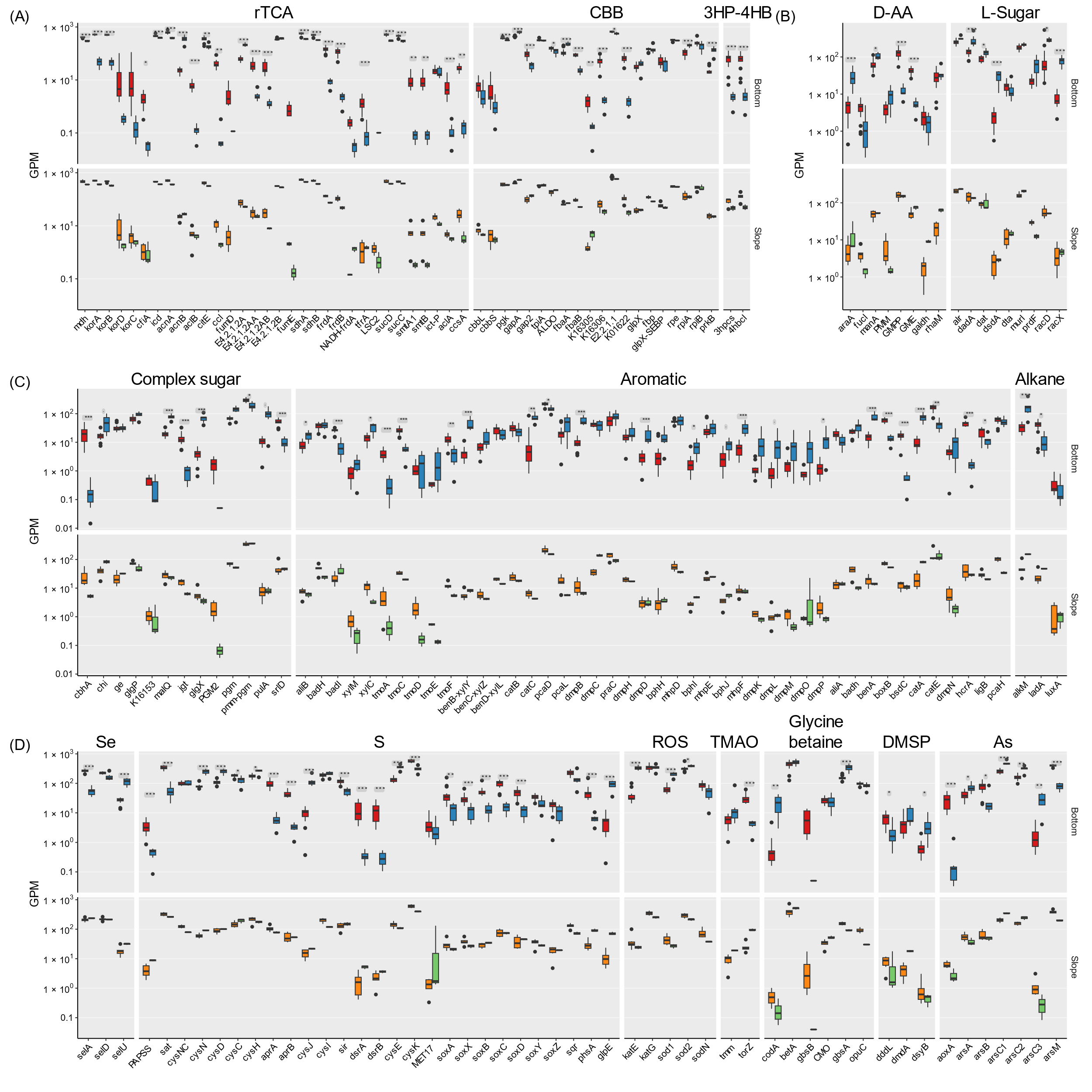
**

## Figure. S6. Distribution of key genes in nitrogen metabolism in MAGs from samples belonging to different sampling groups at class level.

**
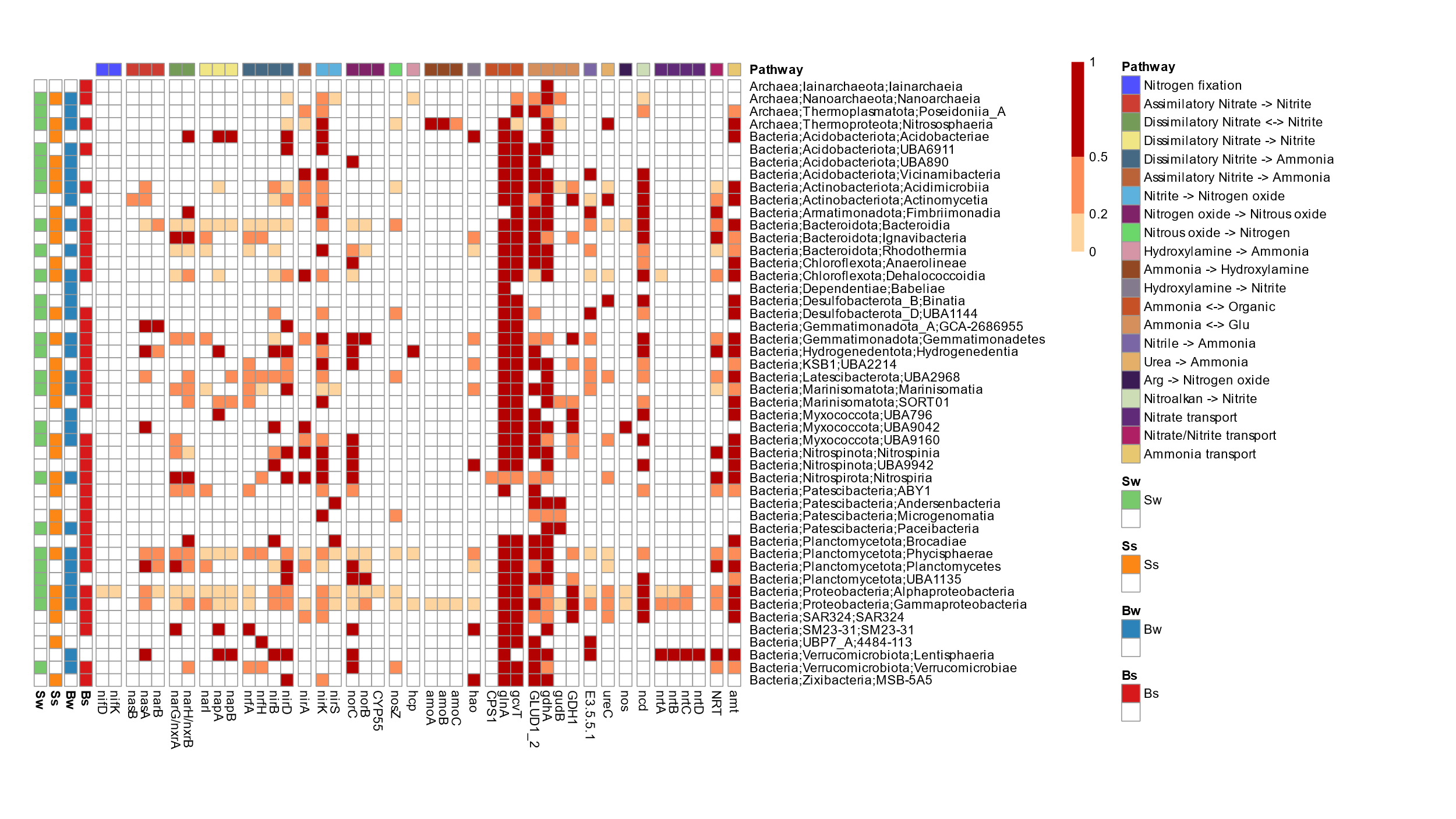
**

## Figure. S7. Co-occurrence network of key genes in nitrogen metabolism of MAGs.

Co-occurrence network was constructed by genes in all MAGs (A), archaeal MAGs (B), and Bacterial MAGs (C) belonging to the groups of slope seawater (Sw), slope sediments (Ss), bottom seawater (Bw), bottom sediments (Bs).

**(A) all MAGs**

**
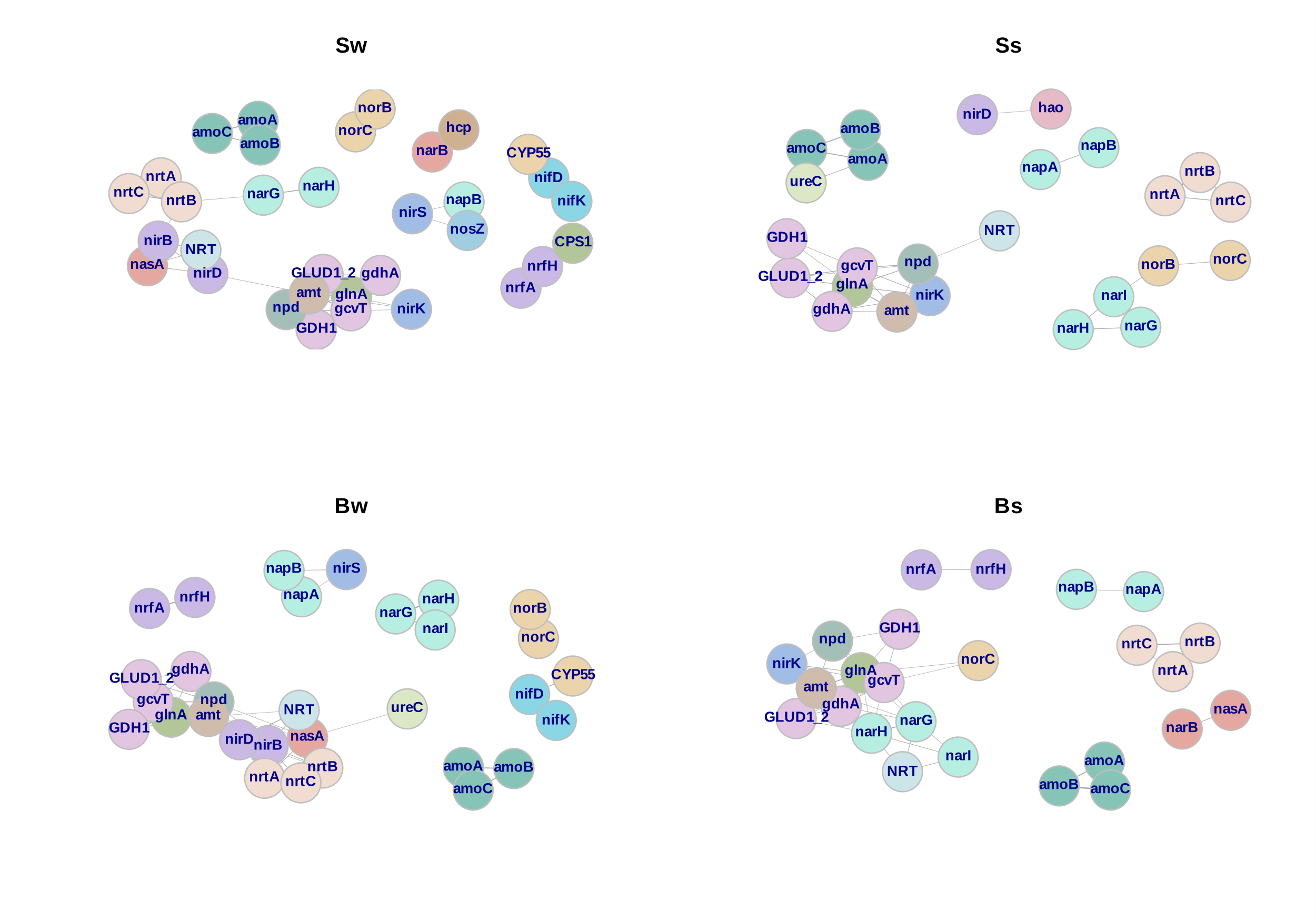
**

**(B) Archaeal MAGs**

**
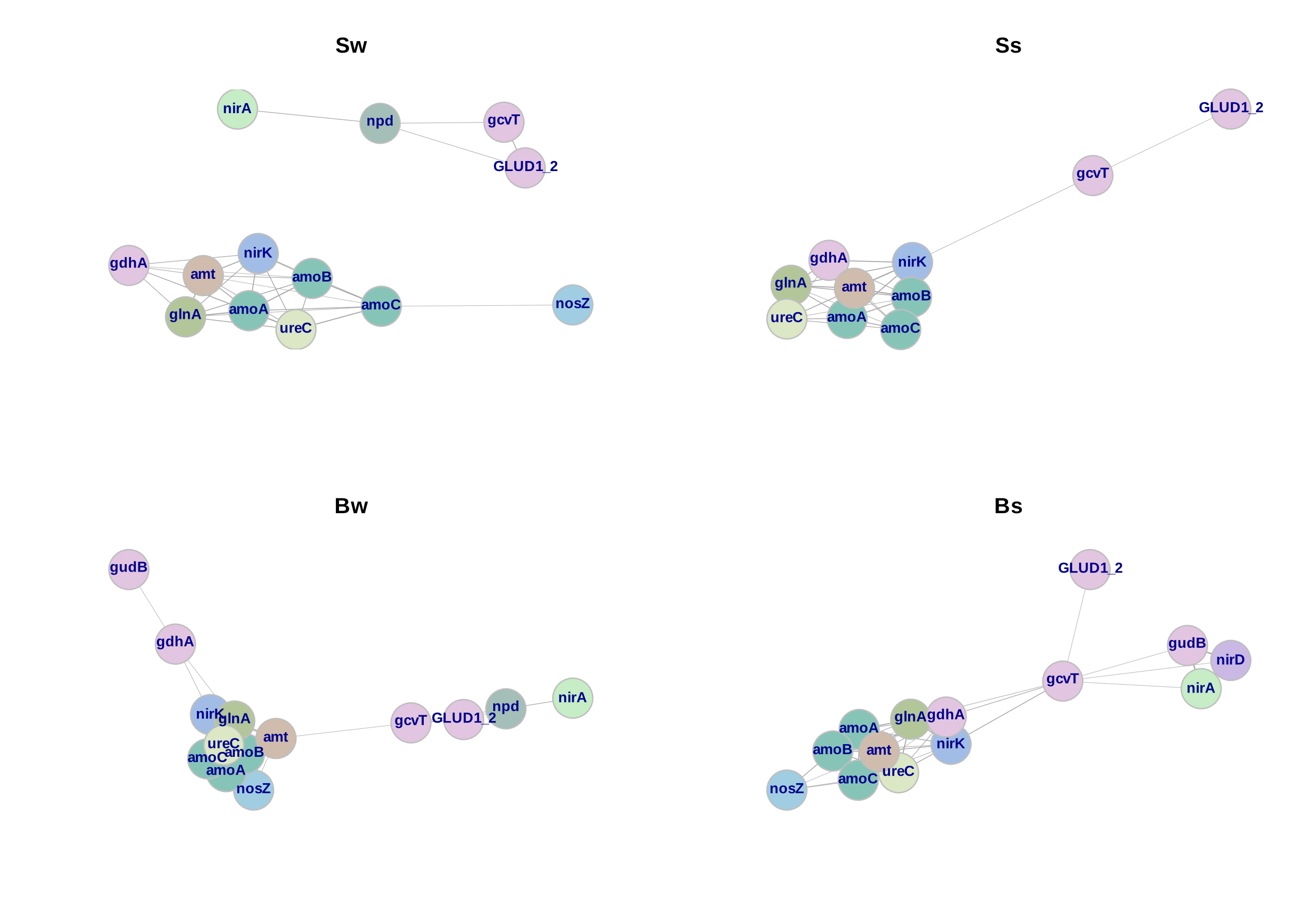
**

**(C) Bacterial MAGs**


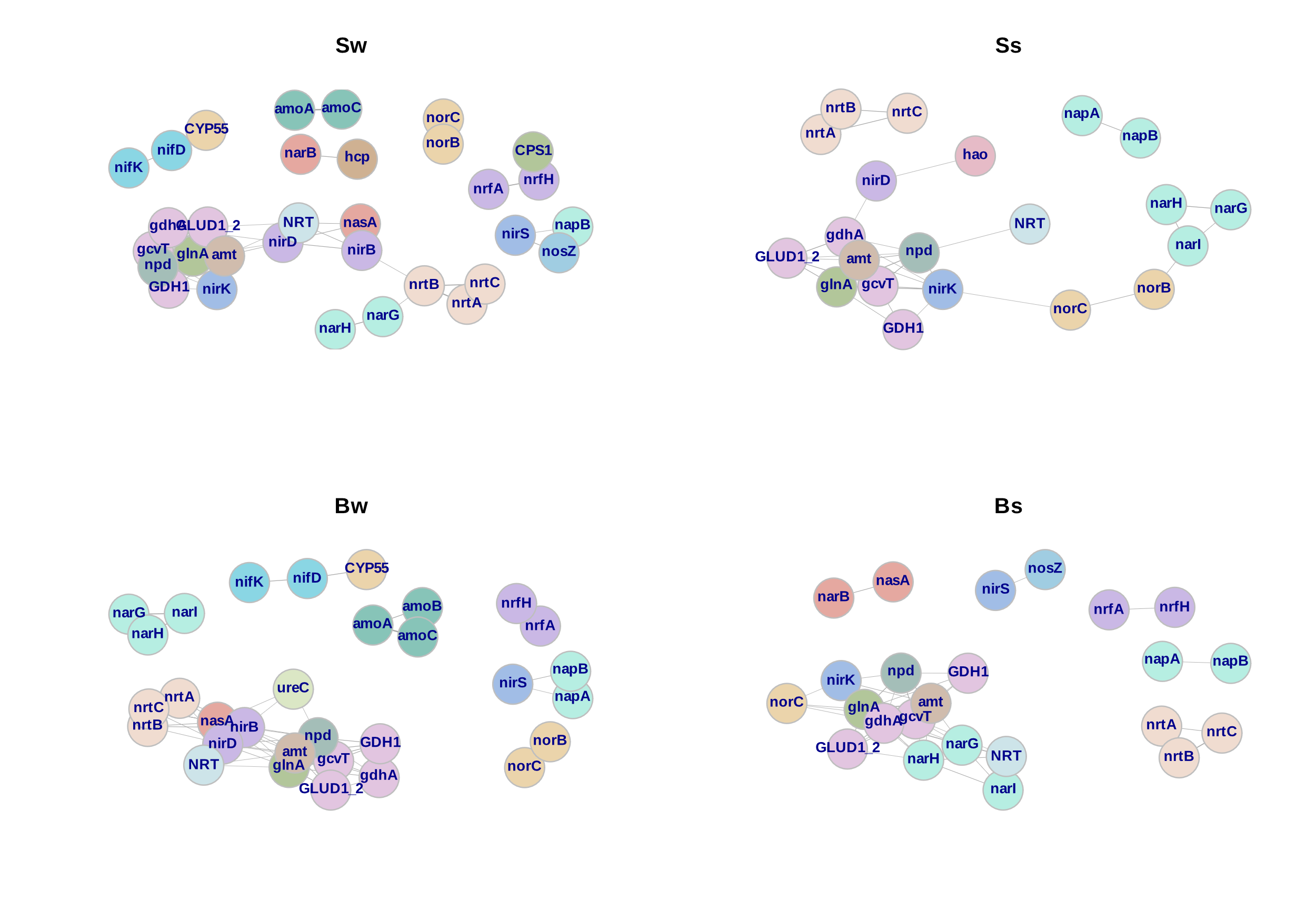


## Figure. S8. Relative abundance and phylogeny of *Nitrososphaeria* MAGs and genes.

MAGs of *Nitrososphaeria* are used for detailed description. (A) Key gene abundance (GPM) and genome abundance of *Nitrososphaeria* MAGs, (B) phylogenic tree of MAGs with *amoB* gene annotated.

**(A)**

**
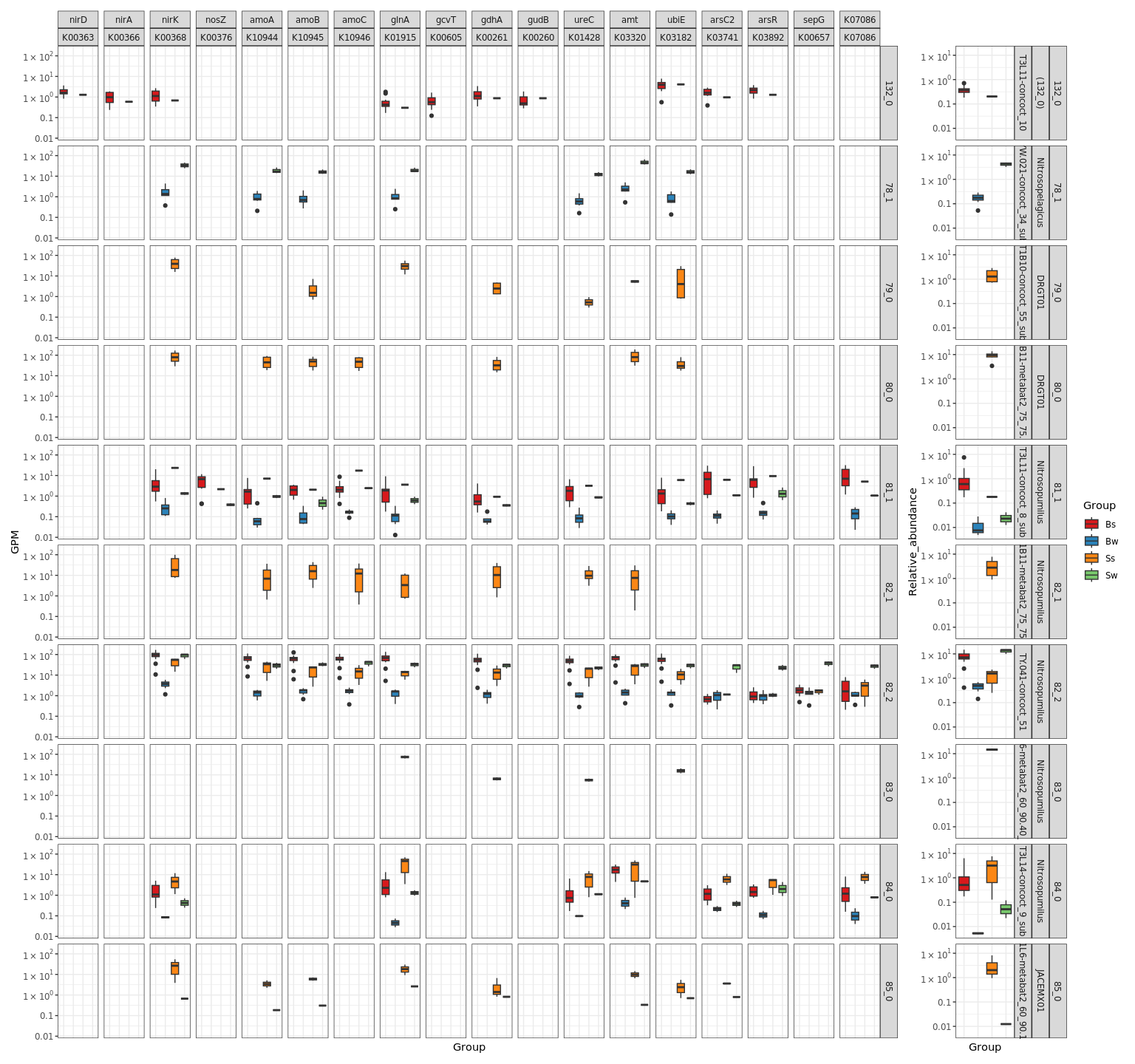
**

**(B)**


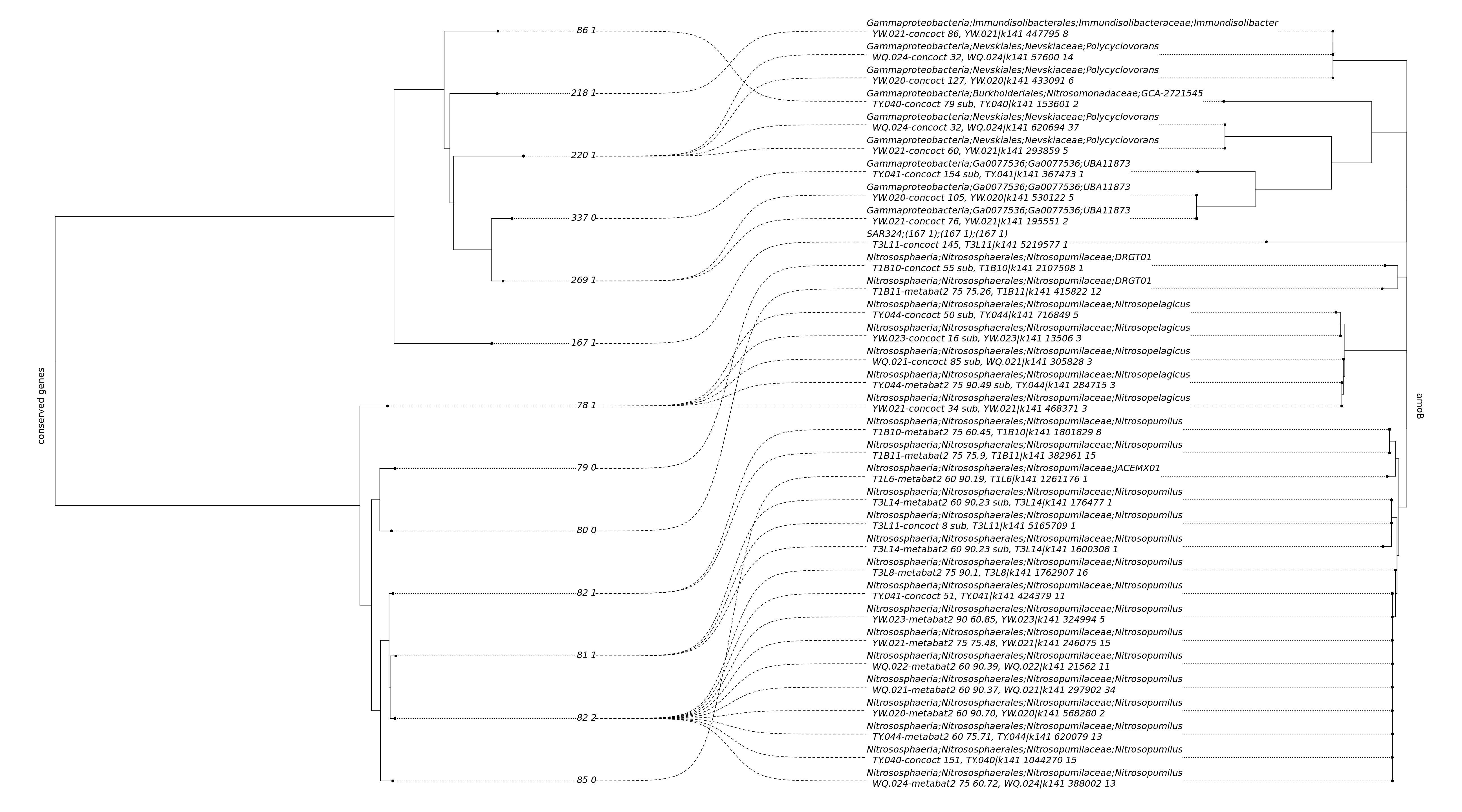


## Figure. S9. Proportions of five ecological processes driven the community assembly in single environment or between two environments.

The percentage of relative importance of driving forces for the prokaryotic community between two environments according to 16S rRNA results and metagenomic results.

**
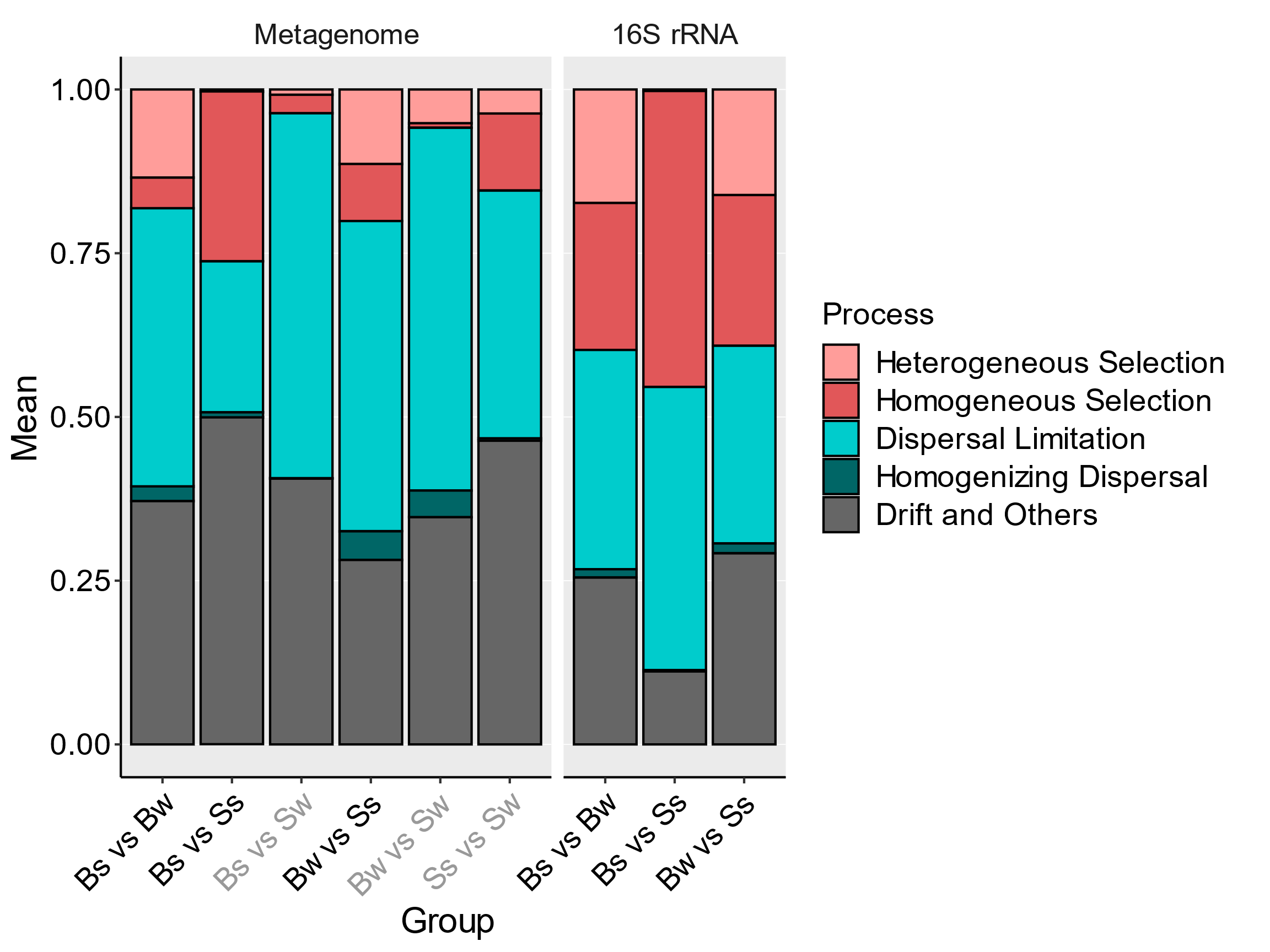
**

## Figure. S10. Trend of population exchange between samples.

The community exchange was calculated by flux (trend of directional movement) and mean (degree of community exchange). The exchange between samples could be classified as four types.


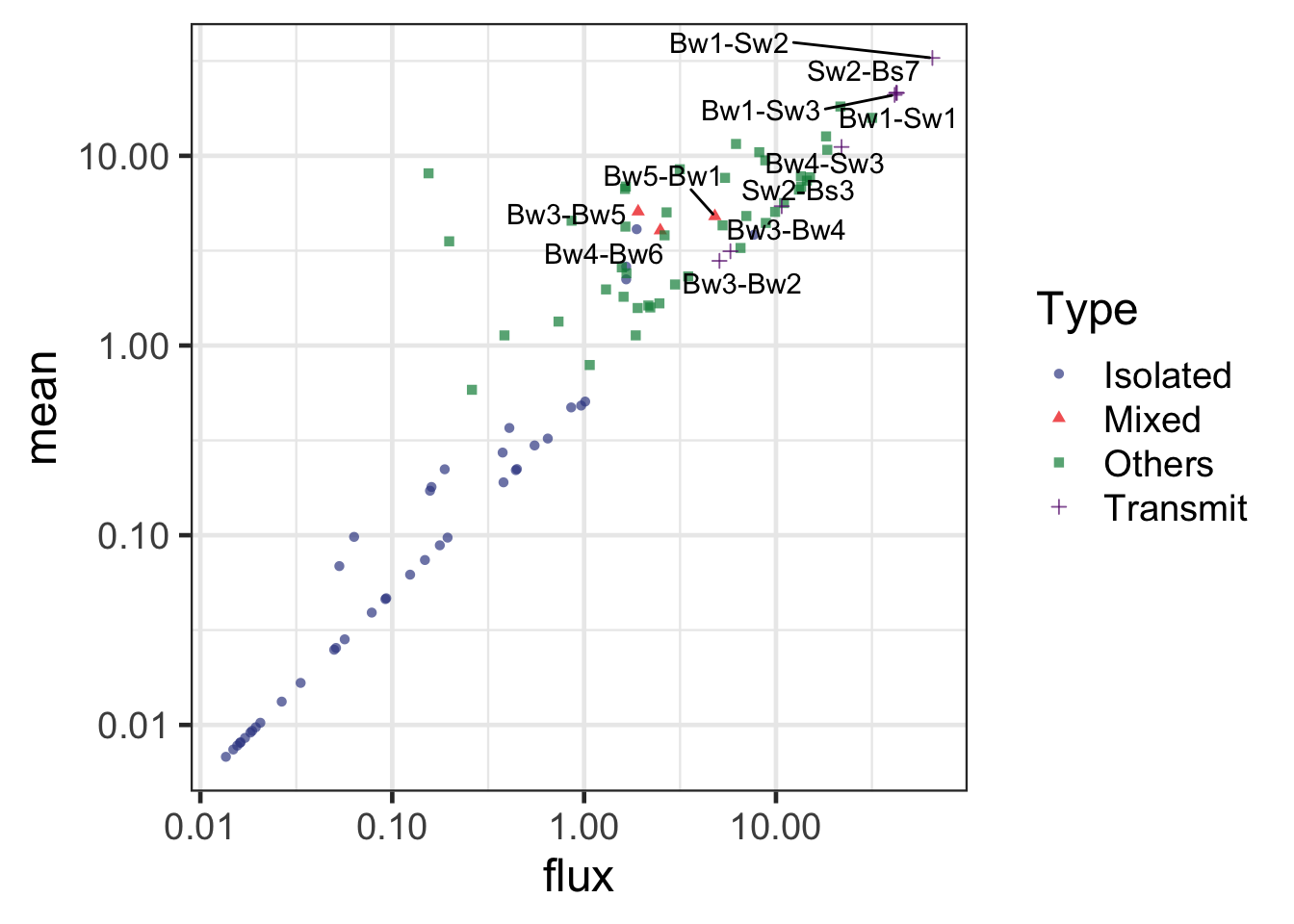


# Supplemental Datasets and Tables

## Supplemental Table S1. Environmental parameters for seawater and sediment samples from the Challenger Deep used in this study.

## Supplemental Table S2. Relative abundance of class-level clades in samples from bottom seawater, bottom sediments, slope seawater and slope sediments.

## Supplemental Table S3. Representative gene list and relative abundancy of genes in N metabolism among all clean reads in different samples.

## Sheet 1: Representative gene list involved in carbon, nitrogen, sulfur and heavy metal metabolism among all clean reads in different samples.

## Sheet 2: Relative abundancy of genes involved to N metabolism among all clean reads in different samples.

## Supplemental Table S4. Description and information of all MAGs beyond middle-quality in this study.

## Sheet 1: Genomic features and taxonomy of MAGs.

## Sheet 2: KO annotation in MAGs.
